# Adenoviral vector oropharyngeal spray immunization elicits mucosal immunity and protects against heterologous SARS-CoV-2 infection

**DOI:** 10.1038/s41541-025-01307-6

**Published:** 2025-12-13

**Authors:** Gerrit Koopman, Petra Mooij, Maria Gaudino, Roja Fidel Acar, Pascal Irrgang, Alina Russ, Dafne Blankenstein, Zahra Fagrouch, Daniella Mortier, Kinga P. Böszörményi, Edmond J. Remarque, Willy M. Bogers, Ernst J. Verschoor, Thomas Gramberg, Matthias Tenbusch

**Affiliations:** 1 Biomedical Primate Research Centre, Rijswijk, The Netherlands; 2 Harald zur Hausen Institute of Virology, Uniklinikum Erlangen, Friedrich-Alexander-Universität Erlangen-Nürnberg (FAU), Erlangen, Germany; 3 FAU Profile Center Immunomedicine, Friedrich-Alexander-Universität Erlangen-Nürnberg, Erlangen, Germany

**Keywords:** Viral infection, Viral infection, Live attenuated vaccines, RNA vaccines

## Abstract

Mucosal immunity may be required to prevent the ongoing transmission of SARS-CoV-2 emerging variants of concern. To define the most efficient way to induce protective mucosal immunity, we compared three different forms of mucosal antigen exposure in mRNA pre-immunized rhesus macaques. Two vaccine groups received an oropharyngeal spray immunization with either an adenoviral vector or a live-attenuated SARS-CoV-2 vaccine (LAV). A third group was infected with SARS-CoV-2 Delta variant as a comparator group representing the exposure history of most humans. Profound levels of SARS-CoV-2-specific IgA antibodies and mucosal T cell responses in the bronchoalveolar lavage next to systemic IgG antibodies were induced after the adenoviral vector boost and the delta infection, but not after LAV immunization. Consequently, these two groups were better protected against a challenge infection with an immune-escape variant of the Omicron lineage EG.5.1.1 showing almost no upper and lower respiratory tract infection. The adenoviral vector vaccine would be a promising candidate for booster vaccinations to interrupt ongoing viral transmission and could generate similar levels of protection as a natural encounter with heterologous SARS-CoV-2.

## Introduction

SARS-CoV-2, the virus causing coronavirus disease 2019 (COVID-19), has rapidly spread around the world since its first emergence in 2019, resulting in over 770 million cases and over 7 million estimated deaths as of February 2025 (WHO COVID-19 dashboard, 2025; available from https://data.who.int/dashboards/covid19/cases). Vaccines that are currently licensed and regularly updated for the newly emerging SARS-CoV-2 variants provide excellent protection from severe COVID-19, hospitalization and death^1,2^. However, breakthrough infections have been reported in fully vaccinated individuals and these numbers increased over time due to immune escape variants, such as Omicron, and due to waning population immunity^3,4^. The ongoing risk of infection may result in severe disease in the elderly and immunocompromised people, and the development of post-acute sequela of COVID.

Secretory IgA and tissue-resident memory cells (T_RM_) at mucosal sites have been identified as major players involved in protection against infection through the respiratory tract^5–8^. Unfortunately, such responses are poorly induced by SARS-CoV-2 vaccines administered via intramuscular injection in comparison with levels observed after a natural breakthrough infection^9–12^. Next to natural SARS-CoV-2 infections, intranasal or aerosolized SARS-CoV-2 vaccines have been shown to induce strong mucosal immune responses that provide protection against infection in several animal models^13–17^. More recently, immunogenicity has also been confirmed in humans^18^. Vaccines tested for mucosal application include soluble spike protein, lipid nanoparticle-encapsulated mRNAs as well as several adenovirus vector-based vaccines, including serotype 5 (Ad5), 26 (Ad26) and chimpanzee adenoviruses (ChAd)^8,14,19–22^. In combination with intramuscular mRNA priming, mucosal booster immunization with Ad5- or Ad19a-based vectors induced strong systemic as well as mucosal responses against SARS-CoV-2 spike (S) and nucleocapsid (N) proteins. In mice, these responses proved to be superior to those induced by solely intramuscular or intranasal immunizations alone^23^. A similar strategy was recently evaluated in rhesus macaques, using an intramuscular Ad26-SARS-CoV-2-Wuhan-S prime followed by an intranasal or intratracheal mucosal boost with bivalent Ad26-SARS-CoV-2-Wuhan-S and Ad26-SARS-CoV-2-Omicron-BA.5-S^24^. The intratracheal, but not the intranasal, boost strategy resulted in lower levels of virus replication in the nose and broncho-alveolar lavage (BAL) compared to intramuscular immunization after challenge with SARS-CoV-2 Omicron BQ.1.1^24^. In another study, mRNA-primed rhesus macaques were boosted with bivalent SARS-CoV-2-Wuhan-S and ChAd-SARS-CoV-2-BA.5-S either by intranasal or aerosol vaccine delivery or intramuscularly^25^. Upon challenge with SARS-CoV-2 omicron XBB.1.16. mucosally boosted animals showed lower levels of virus replication in the nose compared to animals that had received an intramuscular booster, but only a modest reduction of virus replication in BAL was observed^25^.

Besides the use of viral vectors for mucosal immunization, an infection with live attenuated SARS-CoV-2 vaccines (LAV) has also shown great promise in inducing mucosal immunity and protective efficacy in Syrian hamsters^26,27^. Ideally, these mucosal vaccines should generate tissue-localized immunological memory at a comparable level as a SARS-CoV-2 breakthrough infection^28^, and provide broad and durable protection against newly emerging variants. Unlike viral vector vaccines encoding single antigens, LAV express the whole array of viral SARS-CoV-2 proteins, which might result in a broader immune reaction.

In this study, we aim to evaluate different modalities to induce protective, mucosal immunity against heterologous SARS-CoV-2 variants in mRNA-primed rhesus macaques. Specifically, we address the question whether additional booster immunizations provide better protection against newly emerging variants than natural infection events. This gives valuable insights for future vaccine recommendation for the general non-risk population. Here, two vaccine groups receive an oropharyngeal spray immunization with either Ad5 vectors encoding spike and nucleocapsid protein^23^, or a LAV candidate encoding inactivated NSP16 protein, which showed in vitro a tenfold reduced replication compared to the wild-type^29^. All antigens are derived from the Wuhan strain. A third comparator group of mRNA-primed animals is infected with a SARS-CoV-2 Delta variant, which represents the history of a breakthrough infection as seen in a large number of mRNA-vaccinated humans. The protective capacity against infection with a newly emerging SARS-CoV-2 variant is evaluated by challenging all groups with the Omicron EG.5.1.1 strain, which is closely related to XBB.1.9. While the LAV do not induce mucosal antibody or T cell responses in mRNA primed animals, SARS-CoV-2 specific IgG and IgA, and mucosal T cells are present in the two other groups providing protection against the subsequent heterologous infection. As a non-replicating vaccine, the adenoviral vector vaccine might be an excellent candidate for mucosal booster immunizations providing efficient protection against upper and lower respiratory tract infections, at a comparable level as a natural exposure to heterologous SARS-CoV-2. This might be highly relevant to break continuous transmission chains in the case of an ongoing pandemic and should be considered as a concept in terms of preparedness for newly emerging respiratory viruses.

## Results

### Mucosal antigen exposure enhances systemic antibody responses in Comirnaty primed animals

The majority of the human population has already been immunized with a SARS-CoV-2 Wuhan-S vaccine and been exposed to SARS-CoV-2 variants of concern, including the widely spread Delta variant. Mimicking the human situation as much as possible, we immunized (primed) three groups of six animals each twice intramuscularly with the original Comirnaty mRNA vaccine (Supplementary Fig. 1). They were boosted mucosally via oropharyngeal spray with either an adenovirus-5 expressing Wuhan strain S and N (group 1; Ad5), or live-attenuated Wuhan SARS-CoV-2 ΔNsp16 (group 2; LAV) or were infected with a SARS-CoV-2 Delta variant (group 3; Delta). The Comirnaty mRNA primes were given with a 4-week interval and the booster immunization 3 months later (at week 16) following recommended human SARS-CoV-2 immunization schedules. All animals, including a non-vaccinated control group (group 4; control), were challenged 24 weeks after the last immunization with SARS-CoV-2 Omicron EG.5.1.1, which is closely related to XBB.1.9 and XBB.1.5 (Supplementary Fig. 1).

**Fig. 1:**
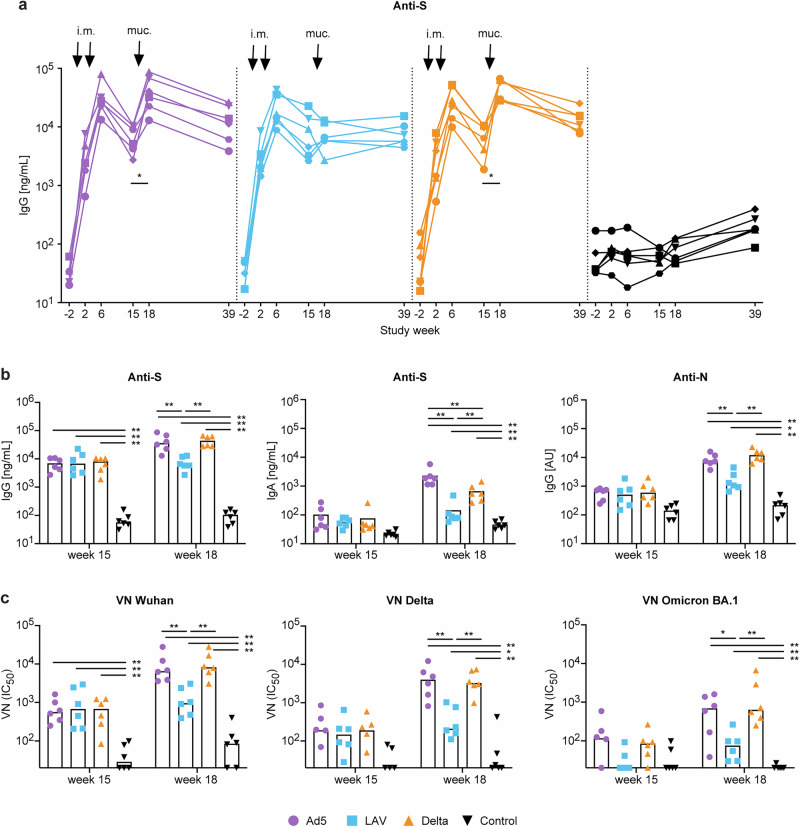
Serum antibody responses. **a** Longitudinal SARS-CoV-2 Wuhan strain Spike-specific IgG antibody responses in serum (in ng/mL) are shown for each individual animal in the Ad5 boosted (in purple), LAV boosted (blue), Delta virus infected (orange), or unvaccinated control animals (black). The two i.m. Comirnaty primes given at weeks 0 and 4 are indicated by arrows i.m. the mucosal booster immunization at week 16 is indicated by arrow muc. **b** Anti-S IgG (in ng/mL, left), anti-S IgA (in ng/mL, middle) and SARS-CoV-2 Wuhan strain anti-N IgG (in Arbitrary Units (AU), right) antibody responses are indicated for each group before (week 15) and after the mucosal boost (week 18). Same color code as in (**a**). **c** Virus neutralization (VN) IC_50_ values were determined at week 15 and 18 against SARS-Cov-2 Wuhan. Delta and Omicron BA.1 pseudotyped viruses. Same color code as in (**a**). Bars represents the median values. Statistical differences between the groups were calculated by Mann–Whitney test, changes in responses over time within one vaccine group were analyzed by a Wilcoxon signed rank test; ^*^p ≤ 0.05. ^**^p < 0.01.

Strong systemic anti-S IgG antibodies were induced in serum by the Comirnaty prime, but these waned over time (1 log reduction within 10 weeks) (Fig. 1a). Antibody levels increased again after mucosal Ad5 booster immunization, and Delta virus breakthrough infection at week 16 (significant increase week 18 versus week 15, Wilcoxon signed rank test *p* = 0.0312), but not after mucosal LAV immunization. Anti-S IgG Ab levels at week 18 were significantly higher in the Ad5 and Delta group than in the LAV group, while there was no difference between these three groups before the boost at week 15 (Fig. 1a, b). All three vaccine groups had significantly higher anti-S IgG levels than the control group, both at week 15 and 18. Systemic anti-S IgA antibodies, and anti-N IgG antibodies were only observed after the mucosal Ad5 boost, and Delta virus breakthrough infection (Fig. 1b), and were in both groups significantly higher than in the mucosal LAV group at week 18 (Fig. 1b). Virus neutralizing (VN) antibodies against the Wuhan strain were induced by mRNA prime in all vaccinated animals, but increased only by mucosal Ad5 boost, and Delta virus breakthrough infection, and not by the LAV boost (Fig. 1c). The VN Ab titers against Delta and Omicron BA.1 were lower than the anti-Wuhan VN Ab titers but showed the same pattern. No cross-neutralization against Omicron XBB.1.5 was detected, which is very closely related to the EG.5.1.1 challenge strain. It can therefore be concluded that there are no measurable neutralizing antibodies against the challenge strain.

**Fig. 2:**
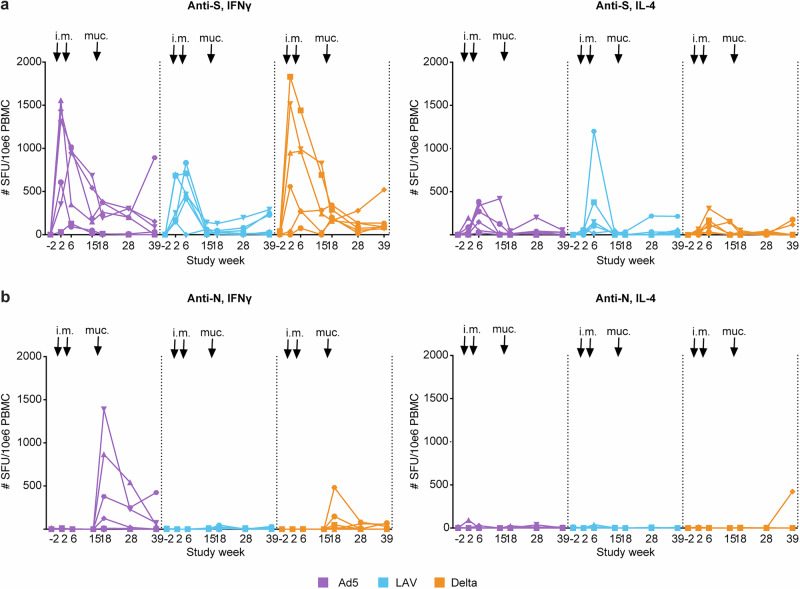
Systemic T cell responses measured in blood. **a** S-specific IFN-γ and IL-4, and **b** N-specific IFN-γ and IL-4 T cell responses. Shown is the number of spot-forming units (SFU) per 10^6^ PBMC in time (study weeks) in Ad5 (in purple), LAV (blue), Delta virus infected (orange), or unvaccinated control animals (black). The intramuscular Comirnaty primes given at weeks 0 and 4 are indicated by arrows i.m., the mucosal booster immunization at week 16 is indicated by arrow muc.

Next to the humoral response, mRNA vaccination induced systemic anti-S T cell responses as determined by ELIspot assays using peripheral blood mononuclear cells (PBMC), in which strong IFN-γ- and modest IL-4 responses were detected after peptide stimulation. These responses waned over time and did not increase after either mucosal Ad5 or LAV boost, or Delta breakthrough infection given at week 16 (Fig. 2a). There were no significant differences between the three vaccine groups in anti-S responses (Mann–Whitney U test). Anti-N IFN-γ ELISpot responses were only apparent after the mucosal Ad5 boost, and in some animals after the Delta virus breakthrough infection, but not after the mucosal LAV immunization (Fig. 2b). Anti-N IL-4 T cell responses were not detected.

**Fig. 3:**
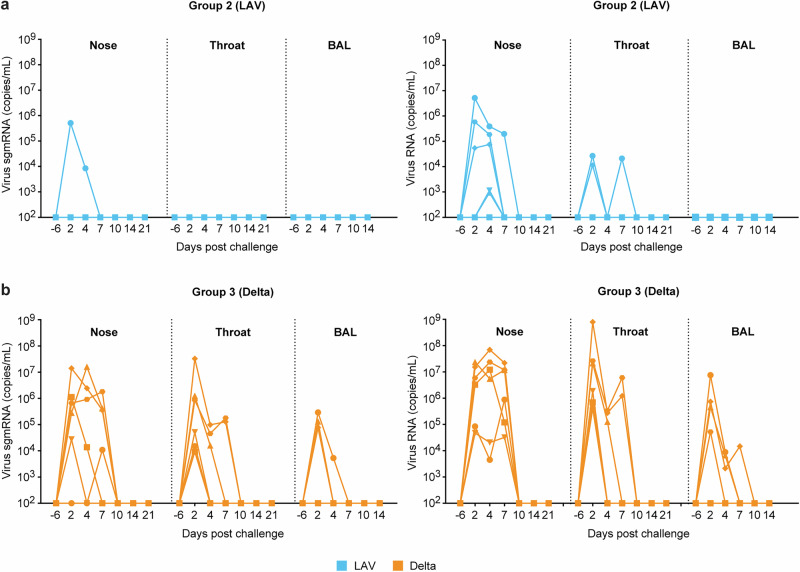
Virus load in mucosal samples after LAV or Delta virus exposure. Shown are for **a** LAV exposed and **b** Delta virus exposed animals the sgmRNA (left graphs) and genomic RNA (right graphs) levels detected in nasal swabs, throat swabs or BAL fluid in time (days) post boost (at week 16).

Since both LAV and SARS-CoV-2 Delta virus are expected to result in a transient infection, nose and throat swabs as well as BAL were collected on days 0, 2, 4, 7, 10, 14, and 21 post exposure. Almost no sub-genomic mRNA (sgmRNA), which is considered as an indicator for active virus replication^30^, could be detected in either nose, throat, or BAL of the LAV immunized animals (Fig. 3a). In contrast, high levels were observed upon exposure to the Delta variant (Fig. 3b). There were no clinical symptoms observed upon Delta virus infection. The fact that genomic RNA (gRNA) was detected in the LAV animals (Fig. 3a, right graph) indicates adequate delivery of the virus. However, the observed RNA levels were much lower than in the Delta group animals (Fig. 3b, right graph). Previous studies in mice and hamsters have shown good replicative capacity and immunogenicity of NSp16-mutant viruses^27,31^. However, the dose of 10^6^ TClD_50_ used in our study may have been insufficient to overcome Comirnaty-induced immunity against the homologous LAV. Such an inhibitory effect was not noted against the heterologous Delta variant, where sgmRNA levels were comparable to levels observed in previous studies in unvaccinated control animals (Supplementary Fig. 4).

### Mucosal immune responses induced by the different boost modalities

**Fig. 4:**
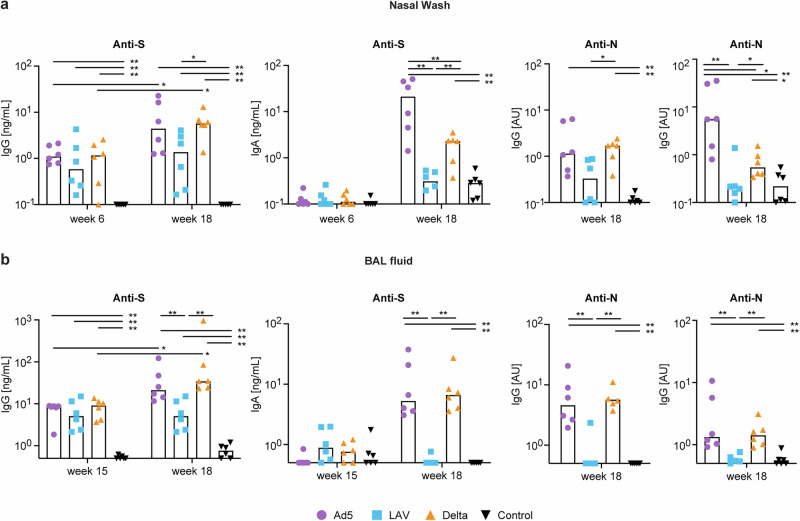
Mucosal antibody responses. **a** SARS-CoV-2 Wuhan strain anti-S IgG (in ng/mL), anti-S IgA (ng/mL), SARS-CoV-2 Wuhan strain anti-N IgG (AU/mL) and anti-N IgA responses (AU/mL) measured at week 6 (2 weeks after the second Comirnaty prime) and/or week 18 (two weeks after the booster immunizations) in nasal washes. **b** SARS-CoV-2 Wuhan strain anti-S IgG (in ng/mL), anti-S IgA (ng/mL), SARS-CoV-2 Wuhan strain anti-N IgG (AU/mL) and anti-N IgA responses (AU/mL) measured at week 15 (11 weeks after the second Comirnaty prime) and/or week 18 (2 weeks after the booster immunizations) in BAL fluid. Responses in Ad5 boosted (in purple), LAV boosted (blue), Delta virus infected (orange), or unvaccinated control animals (black) are shown. Bars represent the median values. Statistical differences between the groups were calculated by Mann–Whitney test, changes in responses over time within one vaccine group were analyzed by a Wilcoxon signed rank test; ^*^p ≤ 0.05. ^**^p < 0.01.

Nasal washes as well as BAL were collected after each vaccination to longitudinally evaluate the induction of mucosal antibody responses. After the two doses of Comirnaty, only low levels of anti-S IgG antibodies were detected in nasal washes collected at week 6 (Fig. 4a) and in BAL collected at week 15 (Fig. 4b) in all three vaccine groups. These responses were significantly increased after both the Ad5 boost, and the Delta breakthrough infection in nasal washes as well as BAL fluid collected at week 18 (compared to week 6 for nasal wash and week 15 for BAL, Wilcoxon signed rank test *p* = 0.0312), while there was no significant increase in the LAV group. At week 18, anti-S IgG antibody levels were significantly higher in the Delta group compared to the LAV group in the nasal wash (Mann–Whitney U test), while in BAL fluid, these responses were significantly higher in both the Ad5 and Delta group compared to the LAV group (Mann–Whitney U test). There were no significant differences between the Ad5 and Delta group in either serum, nasal wash or BAL fluid for anti-S IgG antibody levels. Spike-specific IgA was not observed after Comirnaty prime and only induced in nasal wash and BAL after Ad5 boost as well as Delta, but not after the LAV boost (Fig. 4). Interestingly, anti-S IgA antibody levels were significantly higher in the Ad5 compared to the Delta group in serum and nasal wash, while it did not differ in the BAL samples (Mann–Whitney U test). Anti-N responses were only measured at week 18 after the booster immunizations, because exposure to N only occurred via the immunizations given at week 16. Low levels of anti-N IgG were detected in the nasal washes in all three vaccine groups at week 18 and these were significantly higher in the Ad5 and Delta group compared to the control group (Mann–Whitney U test). Furthermore, in the BAL, significantly higher levels of anti-N IgG antibodies were only observed in the Ad5 boost, and Delta breakthrough groups compared to the control group (Fig. 4b), but not in the LAV vaccine group. At week 18, anti-N IgA was also detected in nasal wash as well as BAL fluid, but only in the Ad5 and Delta groups (Fig. 4). In line with anti-S IgA responses, anti-N IgA levels in the nasal washes of Ad5-treated animals were significantly higher than in the ones of the delta-infected animals, while the IgA levels were similar in the BAL samples.

**Fig. 5:**
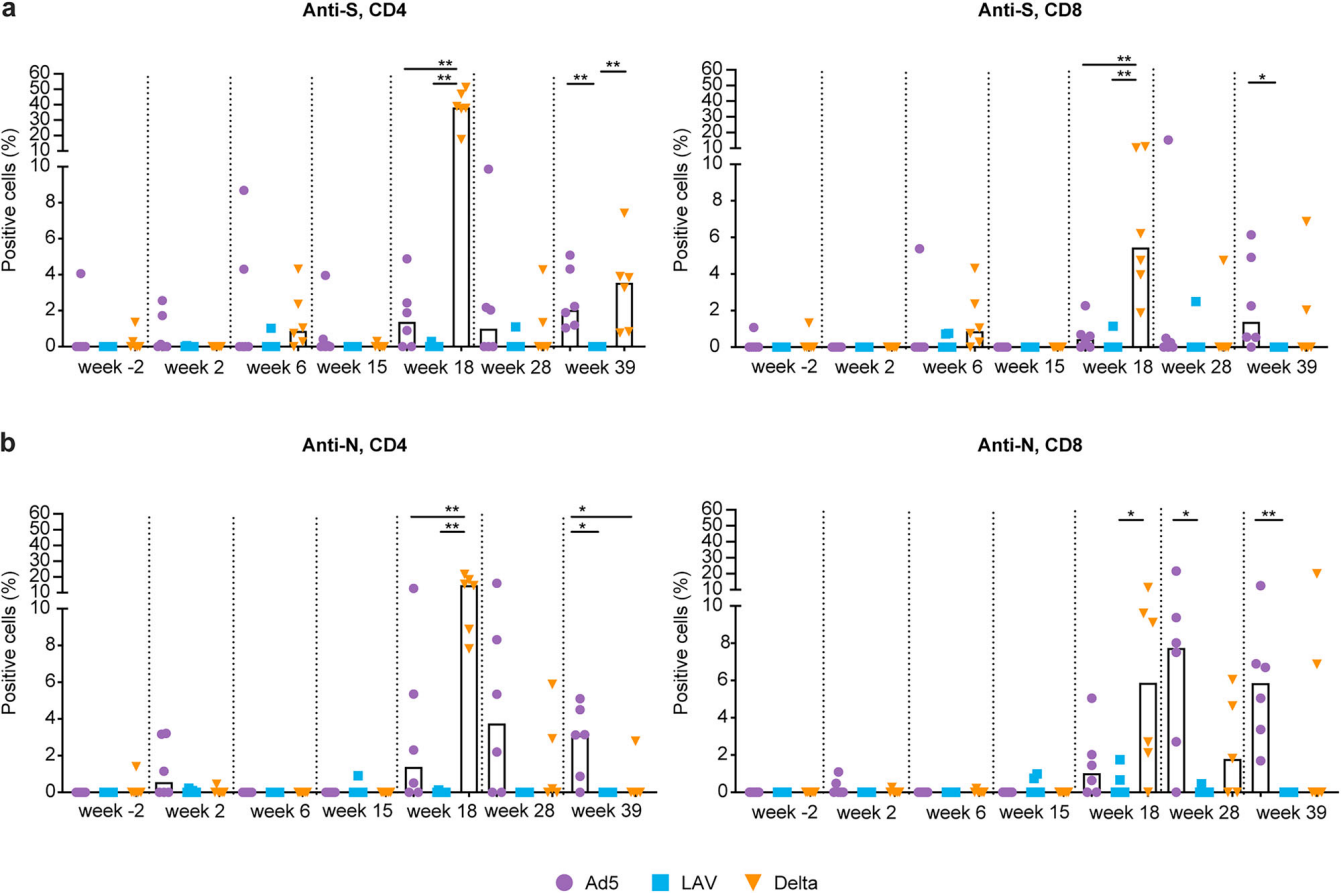
SARS-CoV-2 S- and N-specific T cell responses in BAL cells. **a** Wuhan S-peptide, and **b** Wuhan N-peptide specific, percentage of cytokine (IFN-γ, IL-2, TNFα, IL-4, IL-13, IL-17A) producing CD4 (left graph) and CD8 T cells (right graph) measured after stimulation at the indicated time points (study week) in Ad5 boosted (in purple), LAV boosted (blue), or Delta virus infected (orange) animals are shown. Bars represent the median values. Statistical differences between the groups were calculated by Mann–Whitney test; ^*^p ≤ 0.05. ^**^p < 0.01.

Mucosal T cell responses were measured by intracellular cytokine staining (ICS) performed on cells isolated from BAL fluid. As shown in Fig. 5a, S-specific CD4 or CD8 responses were rarely detected before the booster immunizations. However, S- as well as N-specific CD4 and CD8 T cells were strongly induced by the Delta breakthrough infection and peaked at week 18, 2 weeks after infection. The Ad5 boost also led to increased CD4 and CD8 responses to S and N, but these were significantly lower than in the Delta group at week 18, and LAV immunization did not induce any responses (Fig. 5). Interestingly, the Ad5 vaccine induced responses were maintained at comparable levels up to week 39, which is 1 week before Omicron EG.5.1.1 challenge, while the Delta breakthrough infection induced responses gradually decreased in time to reach similar, or even lower (anti-N CD8 T cell), levels as the Ad5 induced responses by week 39 (Fig. 5). Both CD4 and CD8 T cell responses were highly polyfunctional in the Ad5 as well as the Delta group, with Th2 (IL-4 and IL-13), as well as Th1 (IFN-γ, IL-2, and TNFα), and some IL-17A cytokine producing Th 17 CD4 T cells being present 2 weeks after the boost (week 18) (Supplementary Fig. 5). In CD8 T cells, type 1 cytokines were more dominant, with a large proportion of triple IFN-γ/IL-2/TNFα cytokine positive cells.

### Efficient protection against heterologous SARS-CoV-2 Omicron EG.5.1.1 infection

**Fig. 6:**
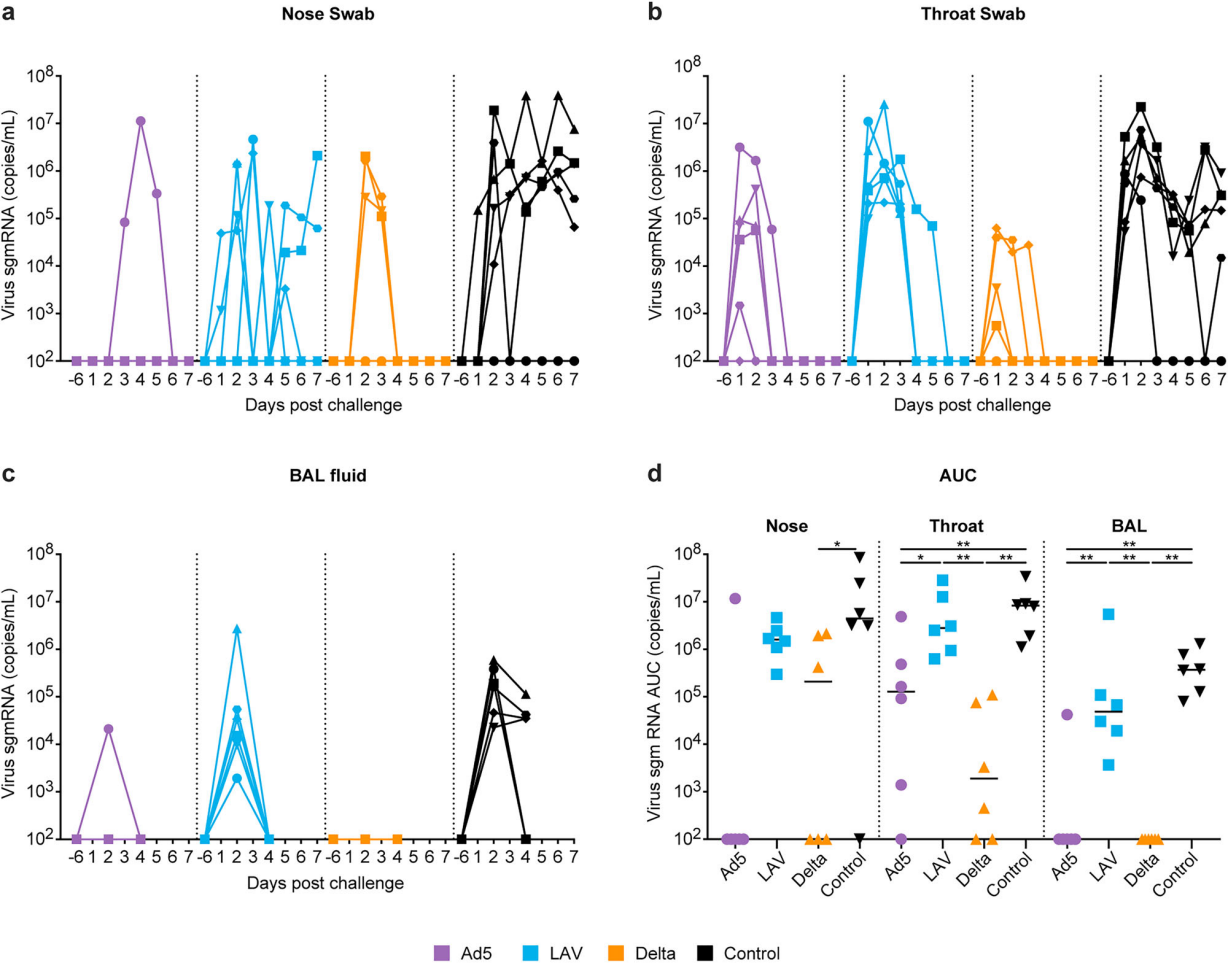
Virus load in nose. throat and BAL measured after SARS-CoV-2 Omicron EG.5.1.1 challenge. Shown are the sgmRNA levels (copies/mL) of each individual animal measured over time (days) after challenge in the Ad5 boosted (purple), LAV boosted (blue), Delta virus infected (orange), or unvaccinated control animals (black). **a** nose swabs, **b** throat swabs, **c** BAL fluid, **d** total sgmRNA in time calculated as area under the curve (AUC) from the time plots shown in Fig. 5a–c. Statistical differences between the groups were calculated by Mann–Whitney test; ^*^p ≤ 0.05. ^**^p < 0.01.

At week 40, 24 weeks after the booster immunizations, all animals were challenged with SARS-CoV-2 Omicron EG.5.1.1 via intranasal plus intratracheal inoculation. In control animals (black symbols), the levels of sgmRNA (Fig. 6) and genomic RNA (Supplementary Fig. 6) in nose, throat, and BAL peaked at day 2 post infection and then stayed at high levels (10^6^ copies/mL) in the nose (Fig. 6a) but decreased by one log in throat (Fig. 6b) and lung (BAL, Fig. 6c) over time. Two out of six control animals cleared virus completely from BAL by day 4, while the other four animals were still positive for viral RNA. Mucosal boosting with the Ad5 vaccine protected five out of six animals from replication in the nose and BAL (Fig. 6a, c, purple symbols) and resulted in faster clearance of the virus in the throat (Fig. 6b). The Delta breakthrough infection only partially protected from subsequent Omicron EG.5.1.1 infection, as three out of six animals became sgmRNA positive in the nose, while viral RNA was undetectable in the BAL of all six animals (Fig. 6a, c orange symbols). Virus was also cleared faster from the throat in this group compared to control animals. A similar pattern was observed for the genomic SARS-CoV-2 RNA (Supplementary Fig. 6). Total virus production over time, plotted as area under the curve (AUC), was significantly lower in the throat and BAL of both the Ad5 and the Delta group compared to the control group and also compared to the LAV group (Fig. 6d). Due to high variation in virus production in the nose, with one Ad5 boosted animal being virus positive and one control animal being virus negative, this comparison did not reach statistical significance. In contrast, the reduction of virus in the nose of Delta group animals was found to be statistically significant (Fig. 6d). Although the AUC analyzes did not indicate protection after the mucosal boosting with LAV, viral clearance seemed to be accelerated compared to control animals, with six out of six animals and five out of six animals being free of virus RNA in BAL and throat, respectively, at day 5 (Fig. 6, blue symbols).

**Fig. 7:**
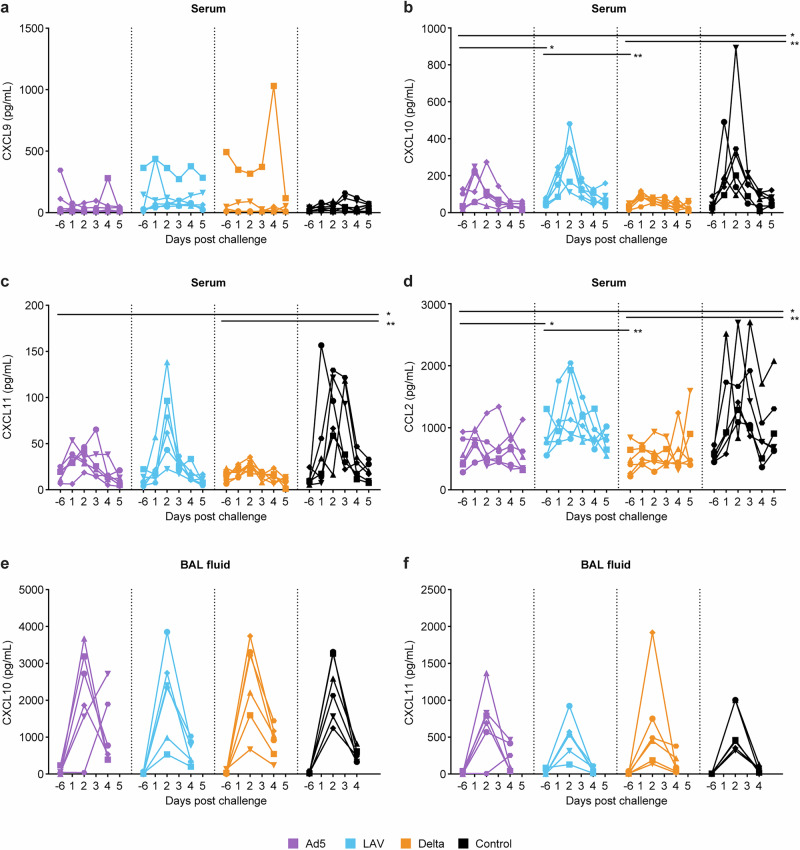
Inflammatory cytokine production measured in serum and BAL fluid after SARS-CoV-2 Omicron EG.5.1.1 challenge. **a**–**d** CXCL9, CXCL10, CXCL11 and CCL2 levels in serum (in pg/mL), and **e**, **f** CXCL10 and CXCL11 levels in BAL fluid, measured over time (days) after challenge are shown for each individual animal in the Ad5 boosted (purple), LAV boosted (blue), Delta virus infected (orange) or unvaccinated control animals (black). Statistical differences between the groups were calculated by Mann–Whitney test using the area under the curve (AUC) calculated for each animal; ^*^p ≤ 0.05, ^**^p < 0.01.

**Fig. 8:**
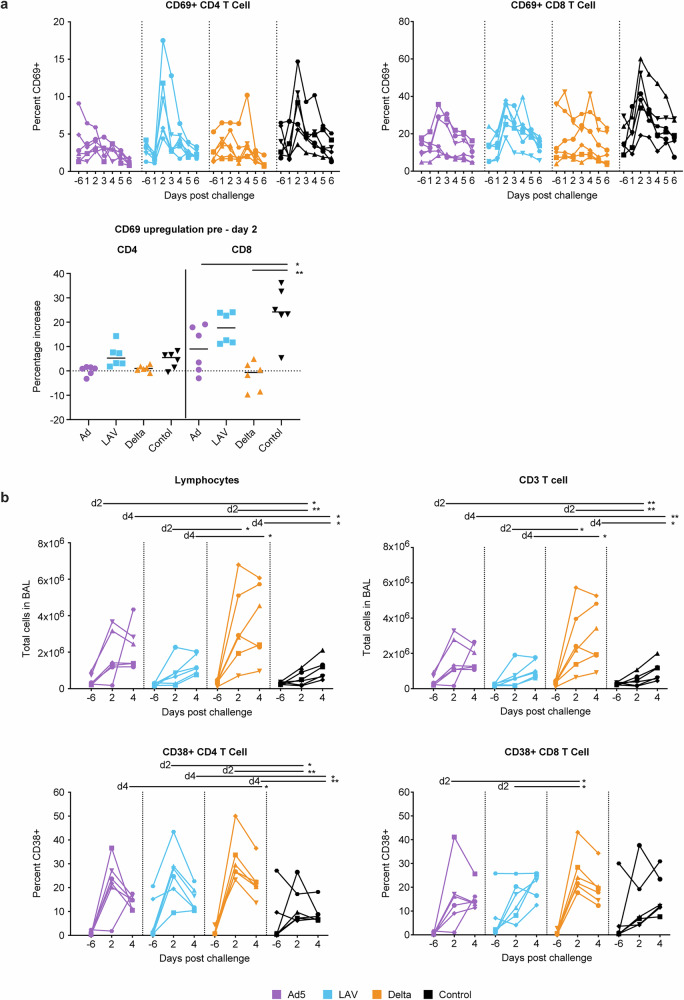
CD69 activation marker expression on peripheral blood T cells and changes in cell number and CD38 activation marker expression on T cells in the BAL. **a** Percentage of CD4 (top left graph) and CD8 (top right graph) T cells in peripheral blood expressing CD69 of each individual animal in time. The lower left graph shows the increase in CD69 expression between day -6 (pre), and day 2 on CD4 and CD8 T cells for each individual animal in the four test groups. **b** total number of lymphocytes present in the collected BAL fluid (top left), total number of CD3 T cells (top right graph), percentage of CD4 (lower left), and CD8 (lower right) T cells in BAL expressing CD38 of each individual animal shown in time. Ad5 boosted (purple), LAV boosted (blue), Delta virus infected (orange), or unvaccinated control animals (black) are shown. Challenge was at day 0. Statistical differences between the groups were calculated by Mann–Whitney test using the values measured for each animal at day 2 (d2), or day 4 (d4) post challenge; ^*^p ≤ 0.05. ^**^p < 0.01.

Protection from heterologous SARS-CoV-2 Omicron EG.5.1.1 infection was also reflected by reduced systemic CXCL10, CXCL11 and CCL2 inflammatory cytokine production in Ad5 (purple symbols), and Delta (orange symbols) animals relative to the control animals, while CXCL9 was rather sporadically induced in single animals independent of the treatment (Fig. 7a–d). In the LAV group these chemokines were not significantly reduced compared to the controls (Fig. 7a–d). Interestingly, the Ad5 and Delta animals also showed significantly lower induction of CXCL10 and CCL2 compared to the LAV group (Fig. 7b, d). In addition, systemic T-cell activation in response to the infection is indicated by upregulation of the CD69 activation marker on CD8 and CD4 T-cells in the control animals (Fig.8a). Here, the increase of CD69+ CD8 cells was significantly less pronounced in the blood of the Ad5 and Delta animals, while the upregulation of CD69 on CD4 T cells did not differ significantly between the groups (Fig. 8a). Other blood leukocyte parameters, such as a transient increase in granulocyte count, lymphocyte count, or transient increase in number of CD16 expressing intermediate monocytes was seen in all vaccine groups to a similar extent as in the control group (Supplementary Fig. 7).

Interestingly, in contrast to our previous studies^32,33^, there was no vaccine-mediated reduction in the local levels of infection-induced CXCL10 and CXCL11 levels in BAL fluid in any of the treated groups (Fig. 7e, f). However, the numbers of lymphocytes and CD3 T cells present in BAL fluid on day 2 and day 4 were significantly higher in the Ad5, and Delta group compared to the control group (Fig. 8b), which may indicate a rapid anamnestic immune response. This could be driven by an influx of reactivated systemic immune cells or the local activation and expansion of resident cells in response to antigen re-exposure by the EG.5.1.1-infected cells. However, the increase is less pronounced in the LAV group, which supports the reactivation of local immunity generated by either the Ad5 booster or the Delta breakthrough. Indeed, an enhanced upregulation of the activation marker CD38 was observed in all vaccine groups on CD4 T cells, but only in the LAV and the Delta group statistically significant higher expression was obtained relative to the controls (Fig. 8b). The CD38 expression on CD8 T cells was less consistent, with a high intra-group variation. Significantly higher CD38 expression was only observed on day 2 after infection in the Delta group compared to the Ad5 and LAV animals. Besides T cells, there was also an increase of the number of B cells, NK cells, and monocytes in BAL fluid after SARS-CoV-2 Omicron infection (Supplementary Fig. 8). The increase in B cells and NK cells was significantly higher in the Delta group compared to the challenge control group, while monocytes were increased at similar levels in all groups (Supplementary Fig. 8).

**Fig. 9:**
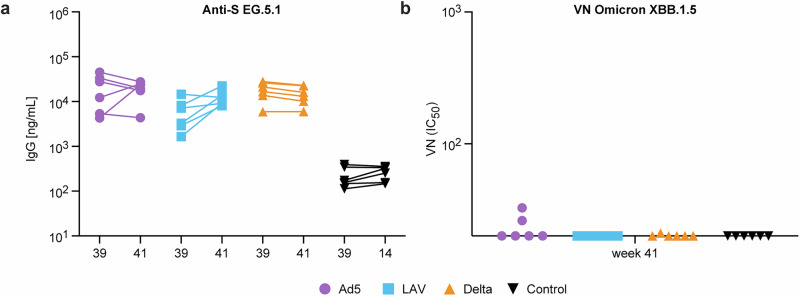
Serum antibody responses before and after SARS-CoV-2 Omicron challenge. **a** SARS-CoV-2 EG.5.1.1 Spike specific IgG antibody responses measured in serum (in ng/mL) at week 39 (1 week before challenge) versus week 41 (1 week after challenge). **b** Virus neutralization (VN) IC_50_ values measured in serum at week 41 against SARS-Cov-2 Omicron XBB.1.5 pseudotyped viruses. Ad5 boosted (purple), LAV boosted (blue), Delta virus infected (orange), or unvaccinated control animals (black) are shown.

Although binding antibodies to the challenge virus EG.5.1.1 were clearly detectable and at comparable levels in all vaccinated animals at the time of challenge (week 39), an increase of these responses after challenge was mostly seen in the LAV treated animals and seems to be related to a higher degree of viral replication (Fig. 9a). A similar increase was not seen in the control group. However, 7 days may have been too short to induce a primary response, while it could have been sufficient to boost the already pre-existing responses in the LAV group. After challenge, only two of the Ad5 and one Delta animal showed a low virus neutralizing titer (respectively 26.1, 32.7 and 21) against Omicron XBB.1.5, which is closely related to the EG.5.1.1 challenge strain (Fig. 9b).

There were no clinical signs or weight loss observed during the Omicron challenge. Histopathological examination of the lungs showed only minimal to mild pulmonary lesions, which were predominantly presented as interstitial inflammation accompanied by bronchiolar, peribronchiolar and perivascular inflammatory infiltrates. There were no differences in histological abnormalities in the lungs between control and vaccinated animals.

### Immune correlates of virus replication after challenge

To identify systemic and mucosal immune parameters associated with reduced viral replication in nose, throat and BAL, a correlation matrix was generated. Virus loads, quantified by sgmRNA values (AUC), were correlated with various immune parameters measured either at the peak of the response at week 18 or 1 week before challenge at week 39. Because of the low number of animals in each vaccine group, analysis was performed for all three vaccine groups together. Systemic and mucosal (lung and nose) anti-S, -N and virus neutralizing (VN) antibodies before challenge negatively correlated with total virus load (AUC) in nose, throat, and lung post Omicron EG.5.1.1 infection (Table 1). This correlation was strongest for antibodies measured directly after the boost (week 18), while it was weaker, or not reaching the *p* = 0.05 threshold at the later time point (week 39). Systemic S- and N-specific IFN-γ, and IL-4 producing T cell responses measured before infection did generally not correlate with virus loads post infection, except for S-specific IFN-γ responses measured at week 18, that showed negative correlation with virus replication in throat and lung (Table 1). Interestingly, mucosal (BAL) S- and N-specific CD4 and S-specific CD8 T cell peak responses (BAL, week 18) correlated with lower virus load in throat and lung (Table 1). However, only N-specific CD4 and CD8 T-cell responses at week 39 were negatively correlated with virus replication in the nose (Table 1). In conclusion, S and N-specific antibodies as well as local T cell responses in mucosal tissues (BAL), may have contributed to the control of virus replication.

**Table 1:** Correlation between systemic and mucosal immune parameters versus total amount of virus replication (AUC of sgmRNA) measured in nose swabs, throat swabs or BAL fluid

	Nose swab (AUC)		Throat swab (AUC)		BAL fluid (AUC)	
	*r*	*p* value	*r*	*p* value	*r*	*p* value
Spike IgG serum wk 39	**–0.51**	**0.030**	–0.35	0.154	**–0.67**	**0.003**
Spike IgG serum wk 18	**–0.72**	**0.001**	**–0.66**	**0.003**	**–0.86**	**<0.001**
Spike IgA serum wk 18	–0.42	0.079	–0.31	0.209	**–0.61**	**0.007**
N IgG serum wk 18	**–0.57**	**0.014**	**–0.64**	**0.005**	**–0.83**	**<0.001**
Spike IgG NW wk 18	**–0.49**	**0.039**	**–0.69**	**0.001**	**–0.68**	**0.002**
Spike IgA NW wk 18	–0.45	0.062	**–0.53**	**0.025**	**–0.62**	**0.006**
N IgG NW wk 18	**–0.49**	**0.038**	**–0.74**	**0.001**	**–0.69**	**0.001**
Spike IgG BAL wk 18	**–0.57**	**0.014**	**–0.64**	**0.005**	**–0.83**	**<0.001**
Spike IgA BAL wk 18	**–0.50**	**0.036**	**–0.72**	**0.001**	**–0.78**	**<0.001**
N IgG BAL wk 18	**–0.59**	**0.009**	**–0.67**	**0.002**	**–0.83**	**<0.001**
VN Wuhan wk 18	**–0.59**	**0.009**	**–0.58**	**0.010**	**–0.80**	**<0.001**
VN Delta wk 18	**–0.61**	**0.008**	**–0.59**	**0.010**	**–0.83**	**<0.001**
VN BA.1 wk 18	–0.43	0.076	**–0.74**	**0.001**	**–0.80**	**<0.001**
Spot Spike IFN wk 39	–0.14	0.569	–0.24	0.334	–0.18	0.475
Spot N IFN wk 39	–0.43	0.076	–0.16	0.531	–0.26	0.298
Spot Spike IL4 wk 39	–0.12	0.643	0.20	0.434	0.21	0.397
Spot N IL4 wk 39	–0.25	0.325	0.10	0.693	0.02	0.945
Spot Spike IFN wk 18	–0.46	0.055	**–0.70**	**0.001**	**–0.75**	**<0.001**
Spot N IFN wk 18	–0.16	0.518	–0.26	0.292	–0.29	0.235
Spot Spike NP wk 18	–0.07	0.775	–0.06	0.824	–0.25	0.315
Spot N IFN wk 18	0.23	0.360	0.15	0.566	0.10	0.702
BAL CD4 Spike wk 39	–0.43	0.074	**–0.56**	**0.016**	**–0.61**	**0.007**
BAL CD4 N wk 39	**–0.53**	**0.032**	–0.24	0.351	–0.41	0.100
BAL CD8 Spike wk 39	–0.41	0.092	–0.04	0.886	–0.32	0.189
BAL CD8 N wk 39	**–0.52**	**0.028**	–0.23	0.353	–0.41	0.095
BAL CD4 Spike wk 18	–0.17	0.488	**–0.65**	**0.004**	**–0.67**	**0.002**
BAL CD4 N wk 18	–0.18	0.463	**–0.58**	**0.011**	**–0.67**	**0.002**
BAL CD8 Spike wk 18	–0.32	0.197	**–0.55**	**0.017**	**–0.61**	**0.007**
BAL CD8 N wk 18	–0.18	0.477	–0.36	0.146	–0.45	0.062

Spearman *r* and *p* values are shown. Spot: results from ELISpot assay performed on blood samples, NW: nasal wash, N: N-protein, VN: virus neutralizing titer measured in serum. Significant values are indicated in bold.

## Discussion

Since mucosal immunity is considered crucial to prevent initial infection by respiratory viruses and not only severe disease, we aimed to investigate which form of mucosal antigen exposure induces the most efficient immune response at the viral entry site in previously vaccinated individuals. Specifically, we addressed the question whether natural infection events, such as breakthrough infections in vaccinated individuals, can provide better protection against emerging variants in the upcoming seasons than repetitive booster immunizations. To this end, we exposed Comirnaty-primed rhesus macaques via an oropharyngeal spray with a non-replicating Ad5 vector vaccine, or a live-attenuated ΔNSP16mut virus (both based on Wuhan strain-derived antigens), and compared the induced immune status to the one observed after a natural breakthrough infection with the Delta variant. This so-called hybrid immunity, induced by a combination of vaccination and natural infection, has been described to provide superior protection against reinfection compared to vaccination alone during the recent SARS-CoV-2 pandemic^9,34^. Although the intramuscular mRNA vaccination induced strong systemic anti-S antibody responses including neutralizing antibodies against the original Wuhan strain, the low neutralizing capacity against the delta variant could not prevent an experimental infection with this variant 12 weeks after the second mRNA dose resulting in robust viral replication in the upper and lower respiratory tract. Similar breakthrough infection has previously also been described in Comirnaty immunized humans^35^. Consequently, these animals developed very rapidly strong mucosal CD4 and CD8 T cell responses against the S and the N protein as detected by ICS in BAL cells. In addition, S-specific IgA and IgG antibodies were elevated in serum, nasal washes, and again in BAL fluid. This is in line with temporal increases in mucosal IgA antibodies seen in humans after natural breakthrough infections^9,36^.

In sharp contrast, our LAV ΔNSP16 candidate, lacking a functional NSP16 protein, failed to induce substantial mucosal immune responses in the mRNA primed macaques after oropharyngeal spray application, and consequently, did not induce substantial mucosal immune responses. Since we and others have confirmed infectivity and immunogenicity of such ΔNSP16-LAV candidates in mice and hamsters after intranasal delivery^27,31^, it is very likely that the pre-existing immunity induced by the mRNA prime might be the cause for this failure. Specifically, strong neutralizing capacity against the Wuhan strain was detected in all mRNA-vaccinated animals before the mucosal antigen exposure took place. Since the spike protein on the surface of the LAV is identical to the Wuhan variant, neutralizing antibodies most probably prevented the initial infection by the LAV and thereby also subsequent replication, as indicated by the low levels of sgmRNA in the mucosal samples. However, the immunogenicity of LAVs heavily relies on the infection and attenuated replication, because otherwise the low number of viral particles applied (10^6^ particles corresponding to ~3 × 10^7^ spike-trimers^37^ and a total amount of spike protein of ~30 pg will not be sufficient to boost antibody responses. This highlights a potential limitation of homologous LAV approaches in antigen-experienced individuals. Similar observations have been made with licensed live-attenuated influenza vaccines, which were more potent in antigen-naive children than in antigen-experienced adults^38^. However, the impact of pre-existing immunity on the immunogenicity of the LAV candidate might depend strongly on the vaccine used to prime the individual and the degree of attenuation of the respective LAV. Along these lines, LAV have been proven effective against SARS-CoV-2 infection in various animal models, partially even as a booster vaccine in pre-immune animals^26,31^. Since the spike protein is not present on the adenoviral particle, pre-existing antibodies do not impact the transduction efficacy of the Ad5 vector vaccine, and the spike protein can be efficiently expressed in the target cells and boost the antigen-specific memory response. Accordingly, our oropharyngeal spray application of Ad5 vectors encoding S and N proteins as a booster immunization induced strong mucosal anti-S IgA and anti-N IgG and IgA Ab responses, and T cell responses in the BAL, which were later found to negatively correlate with replication of the Omicron challenge virus in throat and BAL.

Interestingly, SARS-CoV-2 Delta breakthrough infection did induce significantly higher anti-S and anti-N CD4 and CD8 T cells responses in BAL cells in the acute phase of the breakthrough infection. However, these responses seemed to be more transient, and long-term memory cells were more sustained after the Ad5 boost. Unfortunately, we could not address the mechanism behind this observation within our study, because this would need detailed analyzes on temporal and spatial expression levels requiring repetitive, invasive tissue sampling. However, the dynamics of the antigen-specific T-cell response could be influenced by several factors, such as antigen expression levels, duration of antigen expression, cell types expressing or presenting the antigens and inflammatory conditions^39^. All these factors might be substantially different in the case of an acute viral infection setting (here: Delta) with antigen amplification and cell lysis during viral replication and the application of a replication-deficient, viral vector. Interestingly, the systemic anti-S T-cell response was neither boosted by the delta infection nor by the mucosal application of Ad5-S. In contrast, N-specific T-cells in PBMC could be identified by ELIspot after the mucosal boost. However, we do compare a recall response to S with a primary response to the N antigen, which might impact the distribution of the activated T-cells as well as the expansion capacity of memory cells versus naive T-cells.

Neutralizing antibodies have been defined as major correlate of protection for SARS-CoV-2 in humans, specifically for protection against vaccine-related variants. In our vaccine groups, we clearly induced neutralizing antibodies against the Wuhan strain, with some cross-neutralization potential against Delta and Omicron BA.1, but not against the heterologous challenge virus EG.5.1.1. Again, the antibody responses were boosted by the Delta infection and the mucosal Ad5 vaccination, but not in the LAV group. Although the spike protein of the Ad5 booster and the delta infection were different, the quantity and the breadth of the anti-S antibody seem to be very comparable between these two groups. Potentially, the phenomenon of original antigenic sin may have affected induction of responses after Delta virus exposure, although antigenic sin has so far been most convincingly shown for heterologous exposure to Omicron variants^40,41^.

However, the binding antibody response to EG.5.1.1 directly before the challenge was very similar in the vaccinated groups and might not explain the different protective capacities. Furthermore, anamnestic antibody responses to EG.5.1.1 were more readily seen in the LAV group, which were marginally protected against infection and showed a higher degree of viral replication than the two other vaccine groups. The two vaccine groups, Ad5 and Delta, with substantial mucosal immunity had significantly lower viral loads in the upper and lower respiratory tract than the control animals or the LAV group. Since none of the animals had detectable neutralizing antibodies against the challenge virus, the initial infection rate should be rather comparable between the groups and a more rapid control of viral replication by the activation of local (memory) responses is the most plausible explanation for the different challenge outcome. Along this line, the levels of inflammatory chemokines in the lungs in the Ad5 and Delta groups were comparable to the LAV and control group (Fig. 7e), despite minimal detectable viral loads. One explanation could be that local, immediate CXCL10 secretion is driven primarily by sensing of the incoming viral particles and/or sensing in the initially infected cells (e.g., Type I IFN), while increased serum levels of CXCL10, CXCL11 or CCL2 indicate a more systemic immune activation, which is less pronounced in the Ad5 and Delta group. This would also be in line with the lower activation status of circulating T-cells in these two groups. However, since these chemokines can be produced by IFNγ activated monocyte/macrophages, it could also be postulated that Ad5 vaccine- or Delta virus-induced trained immunity could play a role^42^.

Finally, the mucosal application of our Ad5 vaccine almost completely abrogated viral replication in the nasal compartment, which is line with two other recent reports using SARS-CoV-2 Omicron BQ.1.1, or XBB.1.16^24,25^ as challenge virus. In both studies, animals had received a mucosal booster immunization with a bivalent adenoviral vector vaccine encoding for SARS-CoV-2 Wuhan-S and Omicron BA.5 S, which results in contrast to our study in cross-neutralizing antibodies against the challenge viruses. This might indicate slightly different contributions of the vaccine-induced antibodies to the protection levels seen in the challenge infection. However, in our study, anti-S IgG and IgA Ab levels in serum, nasal wash, and BAL fluid were all negatively correlated with sgmRNA virus production measured during the course of the infection in throat swabs and BAL. Control of SARS-CoV-2 infection in intranasally vaccinated macaques has previously been reported to be independent of neutralizing antibodies and was correlated with vaccine-induced CD8 T cell responses^43^. Furthermore, CD8 T_RM_ cells in the BAL have been shown to be able to provide protection against Omicron XBB.1.5, and BQ.1.1 challenge in the absence of a neutralizing antibody response in K18-hACE2 transgenic mice, and Syrian hamsters^8^. Interestingly, we observed also a negative correlation between S-specific CD8 T cell responses detected in BAL cells and virus production in throat as well as BAL, indicating a possible role of T cell immunity in clearing the virus. These data are in strong agreement with two recent reports^24,25^, although those studies did not address virus replication in the throat. A potentially important role of CD8 T cells in control of SARS-CoV-2 infection has also been reported in CD8 depletion studies in macaques^44–46^. Next to CD8 T cells, mucosal N- and S-specific CD4 T cell responses were negatively correlated with viral loads in the upper and lower respiratory tract. Highly polyfunctional CD4 and CD8 T_RM_ detected in the BAL were also correlated with reduced viral loads in SARS-CoV-2 infected humans, suggesting a cooperative action in viral clearance^47,48^. In line with this latter study, we would also postulate that the protection seen in our study is based on a cooperative action of local immune components (e.g., IgA and T_RM_), leading to rapid activation of an anti-viral environment and early control of virus replication.

To formally prove the contribution of the mucosal booster immunization to protection, an additional group treated only with Comirnaty would have been the best control. However, mRNA vaccination alone had a strong protective effect against infection in the BAL, but there is only a modest reduction in virus replication in the nose as reported by others^24,25,49,50^. Furthermore, the group receiving a LAV mucosal booster immunization could serve as a mRNA-only control, since there was no increase in systemic or mucosal antibody or T cell responses observed in this group and consequentially also no significant reduction in virus replication in the nose, throat, or BAL.

Additionally, we omitted a group receiving a mucosal Ad5 immunization without the Comirnaty prime, because a mucosal immunization-only approach was less effective in inducing mucosal antibody, and T cell responses in mice^23^. In line with our observation, virus replication in the nose was only inhibited after heterologous SARS-CoV-2 challenge using a prime/boost strategy in macaques^25^. In addition, aerosol Ad5 immunization without intramuscular prime was shown to induce lower virus neutralization titers compared to the prime/boost strategy in humans^22^. While an intranasally applied ChAd-S vaccine did effectively protect macaques against lung pathology and significantly reduced sgmRNA levels in the lung tissues and BAL, the reduction in the nose was less robust^14^. Therefore, utilizing the mucosal spray application as a booster in systemically primed individuals seemed to be the most promising approach to induce long-lasting immunity.

In our study, we chose the oropharyngeal application instead of an intranasal spray application, which is used for the licensed Influenza-LAV^38^. The oropharyngeal application might reduce the risk of transfer of the viral vector vaccine, or the encoded antigens to the brain via the olfactory bulb^27,51^. Furthermore, we have previously demonstrated that this application route results in vector-encoded antigen expression in close proximity to the inductive site of the mucosal-associated lymphatic tissue and tonsils enabling effective immune responses against SIV or RSV in rhesus macaques^5,52^.

In conclusion, we have shown that an oropharyngeal spray application of a non-replicating adenoviral vector vaccine induces strong local immunity in the nose and lungs and can protect against upper and lower respiratory tract infection by a highly divergent immune escape SARS-CoV-2 variant. The level of mucosal immunity and protection obtained were comparable to the one seen after a Delta breakthrough infection. Given the strong reduction of challenge virus replication in the nose, this strategy might be of particular interest in the scenario of an ongoing outbreak or pandemic, since it might not only prevent severe disease, but also break transmission chains. Overall, the further development and implementation of mucosal vaccines should be of high priority in terms of preparedness for newly emerging respiratory pathogens.

## Methods

### Animals and ethics statement

The primary objective of this study was to compare three different types of mucosal antigen exposures and define the most effective one in providing protective immunity against subsequent SARS-CoV-2 infection in rhesus macaques (*Macaca mulatta*). Therefore, the primary endpoint of this study was the reduction of viral loads (subgenomic RNA (sgmRNA)) in respiratory tract samples (BAL, nasal, and throat swabs) determined by qRT-PCR. The control group consists of non-immunized, naive rhesus macaques. A power calculation based on viral load data from previous studies showed that a group size of six animals would be sufficient to detect statistically meaningful results in terms of the primary endpoint.

To reduce the risk of any experimental bias, the animals were randomly assigned to the four groups, using the “aselect” function in the Excel program (Microsoft) to generate random numbers. Due to the different treatment schedule, the immunizations and sample collection could not be performed in a blinded manner. However, during the course of the final SARS-CoV-2 challenge, the scoring of the clinical symptoms as a subjective measure was performed by animal care takers blinded for the treatments. During the data collection, all data points and values were included in the analyzes and no outliers were excluded.

All animals underwent a full physical examination prior to entering the study. All individuals were healthy males with normal clinical chemistry and hematology levels and had to be free of pathogens. Further, they were negative for antibodies to simian T cell leukemia virus and simian retrovirus, and negative for binding antibodies to the S protein of SARS-CoV2.

The study was reviewed and approved by the Dutch “Centrale Commissie Dierproeven” (AVD5020020209404-2) according to Dutch law, article 10a of the “Wet op de Dierproeven”, and BPRC’s Animal Welfare Body (IvD). At first vaccination, the age of the animals ranged from 6 to 10 years old, and they weighed 6.9 to 16 kg. Body weight was measured every time the animals were sedated for biotechnical procedures. General behavior and stool consistency were checked daily during the immunization period. During the course of SARS-CoV-2 infection, animals were checked twice a day, and scored for clinical symptoms according to a previously published scoring system (skin and fur abnormalities, posture, eye and nasal discharge, sneezing and coughing, and respiration rate)^53^. A numeric score of 35 or more served as a predetermined endpoint and justification for killing of the animals. Prior to all experimental procedures, including blood take, injection, tonsillar spray, infection, swabs, BAL collection, animals were sedated by intramuscular injection of ketamine (5 mg/kg) and medetomidine hydrochloride (Cepetor®) (0.05 mg/kg) to induce further sedation and muscle relaxation. At the end of the procedure Atipamezol hydrochloride, 0.5 mg/kg (Revertor®) was used for faster recovery. For euthanasia, the monkeys were first deeply sedated by intramuscular injection of ketamine (12 mg/kg) and medetomidine hydrochloride (Cepetor®) (0.05 mg/kg) and then euthanized by an overdose of barbiturate (70 mg/kg) intravenously. This procedure was performed by a veterinarian under continuous monitoring of the heart rate.

### Study schedule, vaccinations and Omicron challenge

The study consists of one control group (naive), and three experimental groups (Supplementary Fig. 1). Three groups of six animals each were immunized at week 0 and 4 with a pediatric dose of 10 µg of the Comirnaty vaccine BNT162b2 (Pfizer/BioNTech. Mainz. Germany) encoding the full-length S-protein of SARS-CoV-2 Wuhan strain via the intramuscular (i.m.) route. At week 16, one group of animals (Ad5) was mucosally boosted via oropharyngeal spray with Ad5 (II)-CMV-(TetO)-SARS-CoV-2 S-P2TS and Ad5 (II)-CMV-(TetO)-SARS-CoV-2 NP-P2TS, based on sequences derived from the SARS-CoV-2 Wuhan strain that had been codon optimized, 2×10^9^ infectious units of each vector per animal (provided by Sirion Biotech. Martinsried. Germany). Another group of animals (LAV) was mucosally boosted via oropharyngeal spray with a live attenuated SARS-CoV-2ΔNsp16 mutated virus derived from an early SARS-CoV-2 isolate Pangolin B1 (GISAIDEPI_ISL_273237), 10^6^ TClD_50_ per animal^29^. A third group of six animals (Delta) was mucosally infected with a dose of 10^5^ TCID_50_ hCoV-19/Netherlands/NH-RIVM-27142/2021 Delta variant via a combined intranasal (0.5 mL) and intratracheal (4.5 mL) route. A fourth group of six animals (Control) were not immunized and served as controls. At week 40, 24 weeks after the booster immunization/Delta virus infection, all animals were exposed to 10^5^ TCID_50_ of a SARS-CoV-2 Omicron EG.5.1.1 isolate via the combined intranasal (0.5 mL) and intratracheal (4.5 mL) route. Animals were monitored for virus replication by taking tracheal and nasal swabs and BAL. Rectal body temperature was measured during every sedation. 1 week post infection animals were killed. Organs were processed for the detection of virus and histopathology.

### ELISA and ELISpot assay

Serum samples, nasal washes and BAL fluid were tested for the presence of IgG and IgA anti-SARS-CoV-2 S-protein (Wuhan strain, Expres2ion, Horshom, Denmark; cat no. S2-46A-001) and IgG anti-SARS-CoV-2 N-protein (Raybiotech, Inc. Peachtree Corners, GA, USA; catalogue number (cat.) 230-30164) as described previously^32^, with the following modifications: half area plates were coated overnight at 4 °C with 1 µg/mL recombinant S-protein in PBS or with 0.5 µg/mL recombinant N-protein in NaHCO_3_ (pH9.6) buffer, 50 µL per well. Bound IgG anti-S and anti-N antibodies were detected using Goat anti-Human-IgG (H+L)-HRP (Invitrogen, Waltham, MA, USA). Bound IgA anti-S antibodies were detected using biotinylated Goat anti-Human IgA (1:2000, Mabtech, Stockholm, Sweden), followed by streptavidin-HRP (1:1000, Mabtech). Week 39 and 41 sera were also tested for the presence of IgG against the Omicron EG.5.1.1 S-protein (R&D Systems, Minneapolis, MN, USA, cat no. 11451-CV-100). Antibody levels were calculated either in ng/ml when a standard with known concentration was used or in Arbitrary Units (AU) when a positive control serum was used; AU being based on the dilution that results in an OD_450_ value of 1 above the background.

Detection of specific IFN-γ and IL-4-secreting cells was performed by ELISpot assay (U-CyTech, Utrecht, Netherlands), as described previously^33^.

### Virus neutralization assay

Neutralization of the Wuhan, Delta, Omicron B1 or Omicron XBB.1.5 variant was assessed with the help of spike-pseudotyped simian or human immunodeficiency virus particles as described before^54,55^. To produce pseudotyped reporter particles, HEK293T cells were transfected with a SIV-based self-inactivating vector encoding luciferase (pGAE-LucW) plus the SIV-based packaging plasmid (pAdSIV3), or the luciferase encoding vector pCCNanoLuc2EGFP plus a HIV-1-based packaging plasmid (pHIV_NL_GagPol), both combined with the respective spike-encoding plasmid. For the assessment of pseudotype neutralization, HEK293T-ACE2 cells were seeded at 2 × 10^4^ cells per well in a 96-well flat-bottom plate. 24 h later, 60 μL of serial dilutions of the serum samples were incubated with 60 μL of lentiviral particles for 1 h at 37 °C. HEK293T cells were washed with PBS, and the particle-sample mix was added to the cells. 48 h later, medium was discarded, and the cells were washed twice with 200 μL of PBS. After 50 μL of PBS and 25 μL of ONE-Glo (Promega Corp., Madison, USA) were added, and after 3 min. the luciferase signal was assessed on a microplate luminometer (VICTOR X5, PerkinElmer) and analyzed using PerkinElmer 2030 Manager software. The reciprocal serum median inhibitory dose was determined with Prism GraphPad 9 (San Diego, California, USA) by application of the Sigmoidal 4PL function.

### Intracellular cytokine staining of BAL cells

Detection of the antigen-specific cells in freshly isolated BAL was performed by ICS. BAL cells were incubated with anti-CD49d (clone L25, BD Pharmingen, San Diego, CA, USA) antibody (1 µg/mL), and either staphylococcal enterotoxin B (1.25 µg/mL; Sigma, St. Louis, MO, USA), 1 µg/mL SARS-CoV-2 peptide pool covering the S-protein (Miltenyi Biotec, cat. 130-127-953), or N-protein (Miltenyi Biotec, cat no 130-126-699), or medium only at 37 °C for 2 h. Then Brefeldin A (Golgiplug 1:1000, BD Pharmingen) was added to inhibit protein trafficking and cells were incubated for a further 12 h. Cells were then washed with PBS and incubated with 50 µL of live/dead fixable violet dead cell stain kit (Molecular Probes, Eugene, OR, USA, cat. L34955) diluted in PBS for 15 min at room temperature (RT) in subdued light. Next, cells were stained for surface markers by adding 100 µL BD Horizon Brilliant Stain Buffer (BD, cat. 566349) containing a mixture of CD95^BV421^ (clone DX2, Biolegend, San Diego. CA, USA), CD8^BV510^ (clone SK1, Biolegend), CD3^BV650^ (clone SP34.2, BD Pharmingen), CD103^BV711^ (clone BerAct8, Biolegend), CD28^BV785^ (clone CD28.2, Biolegend), CD4^BUV563^ (clone L200, BD Pharmingen), CD279^BUV737^ (clone EH12.1, BD), CD185^PE^ (clone Mu5UBEE, Ebioscience), integrin very late antigen (VLA)-β7^FITC^ (clone FIB504, Biolegend), and CD69^APC-Cy7^ (clone FN50, BD Pharmingen). After 20 min incubation at RT in subdued light, cells were washed with PBS/1% BSA, and subsequently fixed with cytofix/cytoperm fixation and permeabilization solution (BD, cat. 554722) for 20 min at 4 °C. Subsequently, the cells were washed with permeabilization buffer (BD Perm Wash, cat. 554723, diluted 10x in H_2_O), and resuspended in brilliant stain buffer, containing 10% permeabilization buffer, and anti-IL-2^BB700^ (clone MQ1-17H12, Biolegend), IL-4^PE-DazzleC495^ (clone MP4-25D2, Biolegend), anti-TNFα^PE-Cy7^ (clone Mab 11, Biolegend), IL-17A^BV605^ (clone BL168, Biolegend), IL-13^APC^ (clone JES10-5A2, BD Pharmingen), and anti-IFNγ^Alexa700^ mAb (clone B27, BD Pharmingen). After 20 min incubation at RT, cells were washed with permeabilization buffer, then with PBS/1%BSA, and then fixed in 2% paraformaldehyde solution (in PBS) for 16 h. Acquisition was performed on an Aurora fluorescence-activated cell sorting (FACS) machine using company software (Cytek, Fremont, CA, USA) and analyzed using FlowJo software. First, a lymphocyte gate was drawn. Then, singlets were selected using a forward scatter-height (FSC-H)/forward scatter-area (FSC-A) plot, and CD3-positive cells that were negative for the live/dead marker were selected (gating strategy shown in Supplementary Fig. 2). Subsequently, CD4 and CD8 subsets were analyzed for cytokine expression. Boolean gating was used on IFNγ, TNFα, and IL-2 positive cells to analyze the Th1 cytokine expression patterns in detail. Statistical analysis was performed using Bernoulli analysis of subpopulations (*Discrete Distributions: Applications in the health sciences*, Vol. Wiley series in probability and statistics)^56^. To control for possible spill-over between different cytokine markers, -staphylococcal enterotoxin b-stimulated PBMC were stained with the live/dead marker and the complete set of surface markers, followed by an intracellular staining with only one of each of the single cytokine markers.

### FACS analysis blood and BAL cells

Multiparameter FACS analysis was performed to determine the number and percentages of monocytes, T and B-lymphocytes, NK cells, and neutrophilic granulocytes within the leukocyte population as well as the expression of activation markers CD69 and CD38 in blood or BAL cells. The following mAb combination was used for blood cells: Horizon brilliant stain buffer plus (BD, cat. 566385), CD4^BUV395^ (clone L200, BD), CD20^V450^ (clone L27, BD), CD8^BV510^ (clone SK1, biolegend), CD16^BV605^ (clone 3G8, BD), CD3^BV650^ (clone SP34.2, BD), CD45^FITC^ (clone MB4-6D6, Miltenyi), CD14^PE-TxRed^ (clone RM052, Beckman Coulter), HLA-DR^PerCP^ (clone L243, BD), CD159^PE-Cy7^ (clone Z199, Beckman Coulter), CD69^APC-Cy7^ (clone FN50, BD). For staining, 100 µL EDTA blood was incubated with a mAb mix (the optimal amount of each antibody was determined by titration previously) in 5 mL polystyrene round-bottom tubes for 15 min at RT in the dark. Subsequently, 4 mL BD FACS lysing solution (BD, cat. 349202, diluted 10x in H_2_O) was added, followed by incubation at room temperature for 10 min, then centrifugation for 5 min at 500 × *g*. The supernatant was aspirated, and the cells were suspended in 5 mL PBS with 2% formaldehyde, and all samples were stored overnight at 4 °C. Flow cytometry was performed on an Aurora FACS machine using company software (Cytek). For each tube, 100,000 events in the lymphocyte-gate were recorded. For determining the number of granulocytes, lymphocytes, and monocytes within the leukocyte population, all cells that were not debris were selected using a FSC-A/SSC-A dot-plot. CD45^low^/SSC-A^high^ cells were defined as granulocytes. Subsequent gating on the non-granulocyte population was used to identify lymphocyte and monocyte subsets (Supplementary Fig. 3). The non-granulocyte population was further analyzed by plotting HLA-DR against CD3. For the CD3-negative cells, HLA-DR was plotted against CD159a. HLA-DR^pos^/CD159a^neg^ cells were further analyzed in a CD20 versus CD14 plot (Supplementary Fig. 3). Monocytes were defined as HLA-DR^pos^/CD3^neg^/CD20^neg^; B cells as HLA-DR^pos^/CD3^neg^/CD20^pos^, NK-cells as CD3^neg^/CD159a^pos^, and T cells as HLA-DR^neg^/CD3^pos^. Within the monocyte population, a CD14 versus CD16 plot was used to identify classical monocytes (CD14^pos^/CD16^neg^), intermediate monocytes (CD14^pos^/CD16^pos^) and non-classical monocytes (CD14^neg^/CD16^pos^). Total cell numbers were calculated by adding a fixed number of fluorescent beads (Coulter Flow Count fluorophores (cat. 7547053) to the FACS tubes during the incubation and then dividing the number of cells by the number of beads recorded. T cells were subdivided in a CD4 and CD8 single-positive subpopulation, which were analyzed for CD69 activation marker expression.

For BAL cells the following mAb combination was used: Horizon brilliant stain buffer plus (BD, cat. 566385), CD4^BUV395^ (clone L200, BD), CD45^BUV496^ (clone DO58-1283, BD), CD25^BUV737^ (clone 2A3, BD), HLA-DR^BUV805^ (clone L243, BD), CD20^V450^ (clone L27, BD), CD11b^BV480^ (clone IRCF44, BD), CD8^BV510^ (clone SK1, biolegend), CD16^BV605^ (clone 3G8, BD), CD38^FITC^ (clone AT1, Stem cell technologies), CD14^PE-TxRed^ (clone RM052, Beckman Coulter), CD127^PECy5^ (clone eBioRDR5, eBioscience), CD159^PE-Cy7^ (clone Z199, Beckman Coulter), CD3^Alexa700^ (clone SP34.2, BD), CD69^APC-Cy7^ (clone FN50, BD). For staining 300 µL cell suspension was incubated with the mAb mix in 5 mL polypropylene round bottom tubes for 15 min at 4 °C. Then cells were washed with a PBS/1%BSA solution suspended in 5 mL PBS with 2% formaldehyde and stored overnight at 4 °C. As many cell as possible were recorded using an Aurora FACS machine (Cytek). The same gating strategy as described above was used, although CD38 was used as an activation marker since CD69 was already expressed as a T_RM_ marker on a large number of cells. Total cell numbers were calculated based on sample volume and cell counts measured during FACS analysis and recalculated to give total cell numbers in the entire lung aspirate.

### Virus detection

Tracheal and nasal swabs and BAL were analyzed for the presence of corona virus RNA using RT-qPCR as described^33^. SgmRNA was measured according to Wölfel et al.^30^. In brief: viral RNA was isolated using a QIAamp Viral RNA Mini kit (Qiagen Benelux BV, Venlo, The Netherlands) following the manufacturer’s instructions. The RT-qPCR assay was carried out using the Brilliant II QRT-PCR Core Reagent Kit, 1-Step kit (Agilent Technologies BV, Amstelveen, The Netherlands), according to the instructions provided by the manufacturer in a 25 ml volume with final concentrations of 600 nM for both primers, 200 nM for the probe, and 5 nM MgCl_2_, using 10 ml RNA, extracted from 140 mL sample volume. RNA was reverse transcribed for 30 min at 50 °C. Then, after 10 min incubation at 95 °C, the cDNA was amplified for 45 cycles, consisting of 30 s denaturation at 95 °C, followed by a 1-min annealing-extension step at 60 °C. SgmRNA measurement, based on the E-gene, was performed using forward primer CGATCTCTTGTAGATCTGTTCTC, reverse primer TGTGTGCGTACTGCTGCAATAT and 5′-nuclease PCR probe ACACTAGCCATCCTTACTGCGCTTCG. All the reactions were carried out with an iQ5 Multicolor Real-Time PCR Detection System (Bio-Rad Laboratories BV, Veenendaal, The Netherlands). The amount of RNA was quantified based on a standard curve made via in vitro transcription of a synthetic target sequence.

### Cytokines and chemokines

Serum samples, nasal washes, and BAL fluid were tested for the presence of cytokines and chemokines using a LEGENDplexTM NHP Chemokine/Cytokine Panel (13-plex. Biolegend, cat. 740317) assay. The assay was performed according to manufacturer’s instruction with some modifications, as described^33^. The following cytokines were measured: IL-1β, IL-6, CCL11 (Eotaxin), CXCL10 (IP-10), CXCL11 (I-TAC), CCL2 (MCP-1), CXCL9 (MIG), CCL3 (MIP-1*α*), CCL4 (MIP-1β), CCL5 (RANTES), CXCL8 (IL-8), TNF*α*, IFN-γ.

### Statistical analysis and correlations

Statistical significance of differences between the groups in IgG and IgA binding anti-S and IgG binding anti-N levels, neutralizing antibody titers, IFN-γ and IL-4 ELIspot data, ICS data, differences in inflammatory cytokine production, and differences in cell number, and CD69 or CD38 activation marker expression on CD4, and CD8 T cells after SARS-Cov-2 Omicron EG.5.1.1 challenge were calculated by using the Mann–Whitney U test. Changes in responses over time within one vaccine group were analyzed by a Wilcoxon signed-rank test. A two-sided α level of 0.05 was used to determine significance. Differences between the groups in sgmRNA virus levels after SARS-Cov-2 Omicron EG.5.1.1 infection were based on calculated AUC values per animal, and a two-sided Mann–Whitney U test. A correction for multiple comparisons was not made. With the relatively small number of animals this could easily obscure the more subtle differences between the groups (when *p* < 0.05). Furthermore, most of the differences shown here have a *p* < 0.01 and remain significant after correction. Correlations between the different humoral and cellular immune parameters, and total sgmRNA virus levels in time quantified as AUC in nose, throat, or BAL was calculated by Spearman correlation test. *P*-values < 0.05 were considered significant.

## Supplementary information

Supplementary Information

## Data Availability

All data associated with this study are presented in the paper, or the Supplementary Materials. Plasmids, viral vectors, LAV, or other reagents are available upon request to the corresponding author. An excel file containing all the raw data is available on Github: https://github.com/B-P-R-C/2025-CCD028S-Isidore.
