# Supplementary material for: Adenoviral vector oropharyngeal spray immunization elicits mucosal immunity and protects against heterologous SARS-CoV-2 infection
Source: NPJ Vaccines. 2025 Dec 13;10:267. doi: 10.1038/s41541-025-01307-6 (PMC12739146; doi:10.1038/s41541-025-01307-6)
Supplement: Supplementary file 1 — Supplementary Information [file 41541_2025_1307_MOESM1_ESM.pdf]

Supplementary figures

Title: Adenoviral vector oropharyngeal spray immunization elicits mucosal immunity and protects against heterologous SARS-CoV-2 infection.

Authors:

Gerrit Koopman[1*], Petra Mooij[1], Maria Gaudino[1], Roja Fidel Acar[1], Pascal Irrgang[2], Alina Russ[2], Dafne Blankenstein[1], Zahra Fagrouch[1], Daniella Mortier[1], Kinga P. Böszörményi[1], Edmond J. Remarque[1], Willy M. Bogers[1], Ernst J. Verschoor[1], Thomas Gramberg[2,3], Matthias Tenbusch[2,3*]

Affiliations:

[1]Biomedical Primate Research Centre, Rijswijk, Netherlands

[2]Harald zur Hausen Institute of Virology, Uniklinikum Erlangen, Friedrich-Alexander-Universität Erlangen-Nürnberg (FAU), Erlangen, Germany

[3]FAU Profile Center Immunomedicine, Friedrich-Alexander-Universität Erlangen-Nürnberg; Erlangen, Germany

*** Corresponding authors:** e-mail: koopman@bprc.nl (Gerrit Koopman), matthias.tenbusch@fau.de (Matthias tenBusch).

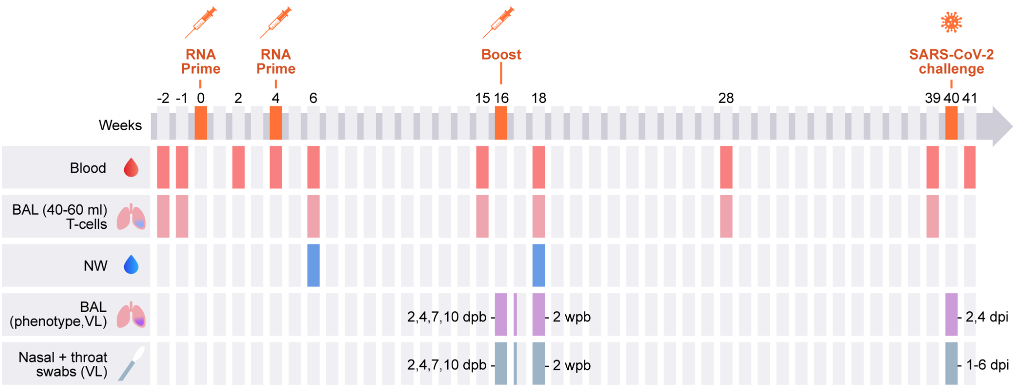

Experimental group	Prime I (W0)	Prime II (W4)	Boost (W16)	SARS-CoV-2 challenge (W40)
**Ad5** N=6	Comirnaty (10µg) (Children dose)	Comirnaty (10µg) (Children dose)	Ad-S, Ad-N (each 1x10^9 IU/spray)	EG.5.1.1 (10^5 $TCID_{50}$) i.n. + i.t.
**LAV** N=6	Comirnaty (10µg) (Children dose)	Comirnaty (10µg) (Children dose)	SARS-CoV-2 ΔNSP16 (10^6 $TCID_{50}$/spray)	EG.5.1.1 (10^5 $TCID_{50}$) i.n. + i.t.
**Delta** N=6	Comirnaty (10µg) (Children dose)	Comirnaty (10µg) (Children dose)	B1.167.2 (10^5 $TCID_{50}$) i.n. + i.t.	EG.5.1.1 (10^5 $TCID_{50}$) i.n. + i.t.
**Control** N=6	PBS	PBS	PBS	EG.5.1.1 (10^5 $TCID_{50}$) i.n. + i.t.

BAL: Bronchoalveolar Lavage; **NW:** nasal wash; **VL:** viral load; **dpb:** days post boost; **wpb:** weeks post boost; **dpi:** days post infection; **W:** week; **i.n.:** intranasal; **i.t.:** intratracheal.

Fig. S1 Study schedule. Three experimental groups of six cynomolgus macaques were immunized at weeks 0 and 4 by the intramuscular (i.m.) route with the SARS-CoV-2 mRNA vaccine Comirnaty and then received at week 16 either an adenoviral vector vaccine or LAV by nasopharyngeal spray or an infection with the SARS-CoV-2 delta variant. All animals, including a group of six unvaccinated controls were challenged at week 40 intranasally plus intratracheally with 1 x 10^5 $TCID_{50}$ of SARS-CoV-2 Omicron EG.5.1.1 variant. Immunizations, challenge and collection of blood, BAL, nasal wash and nasal and throat swabs are indicated in the top graph. Group composition is shown in the table.

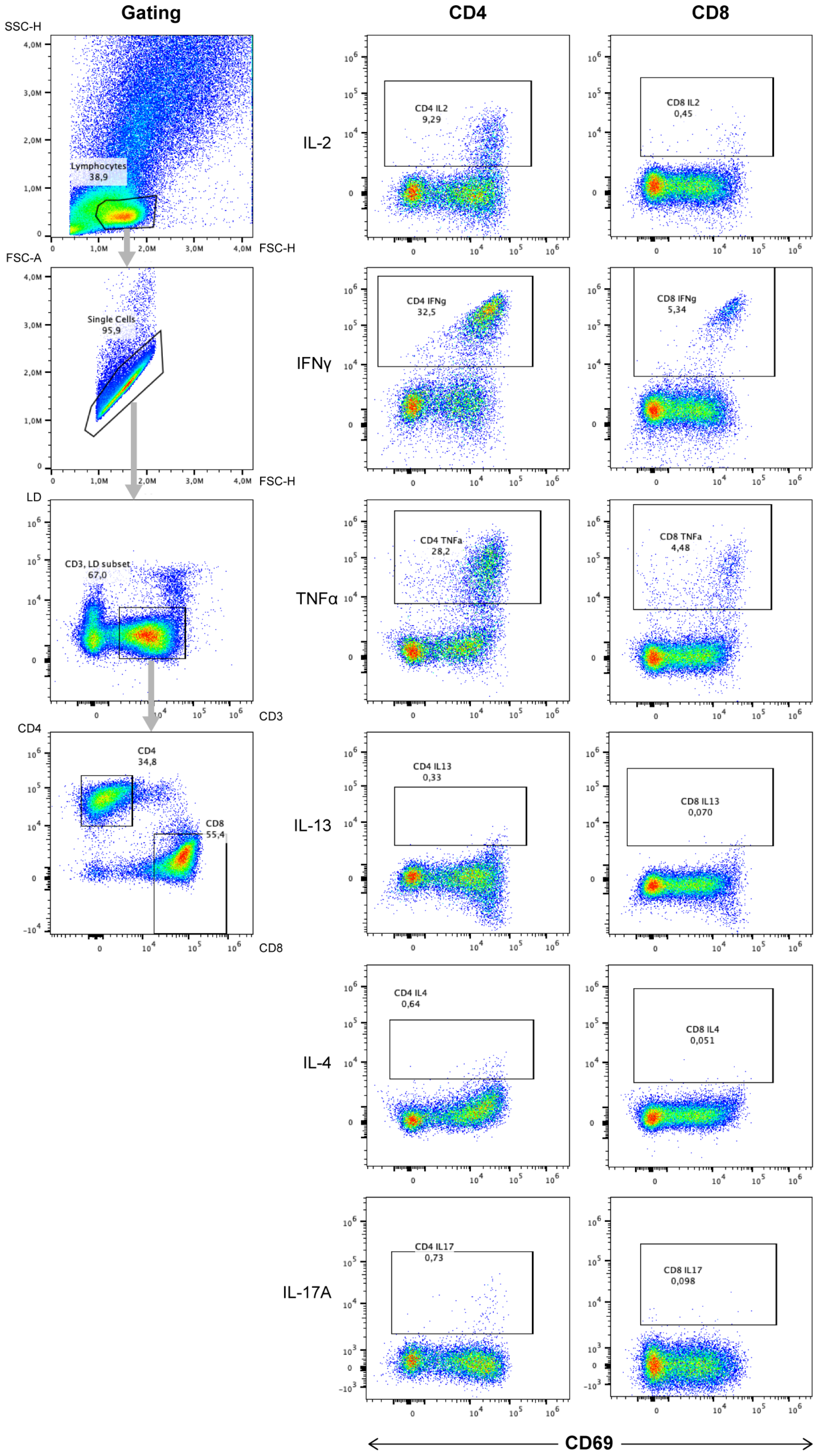

Fig. S2 Gating strategy for analyzing antigen specific cytokine responses in BAL CD4 and CD8 T cells. Shown is analysis of S-peptide pool stimulated cells from an animal (R16063) from the Delta breakthrough infection group, at week 18 (two weeks after infection).

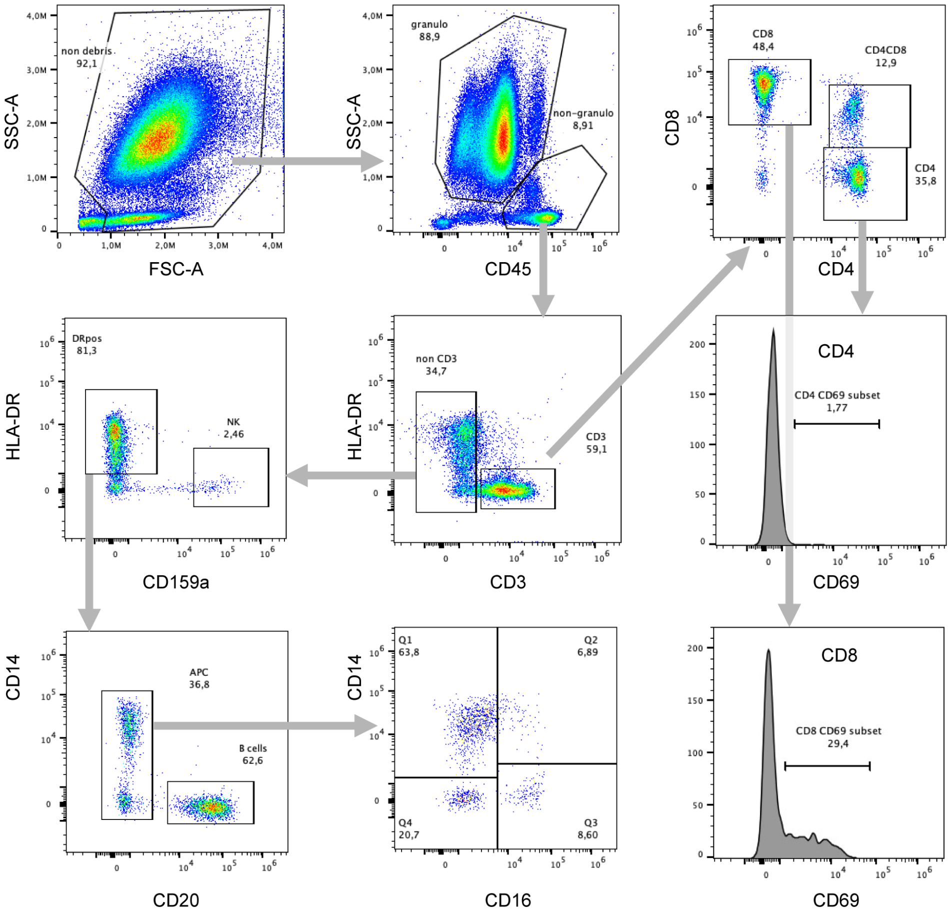

Fig. S3 Gating strategy for analysis of blood samples after SARS-CoV-2 Omicron EG.5.1.1 challenge.

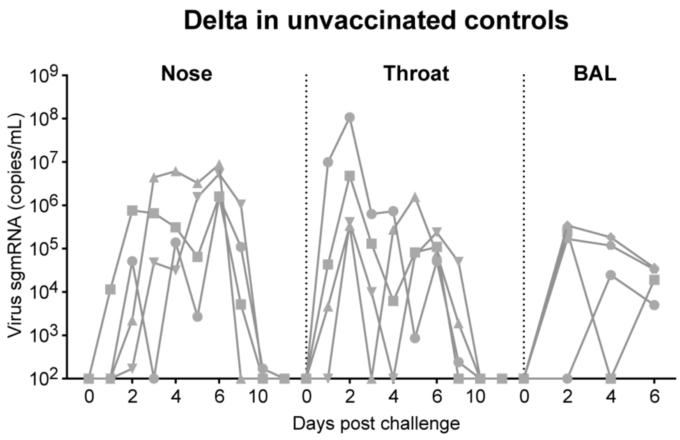

Fig. S4 Virus load in mucosal samples after Delta virus infection in unvaccinated control animals. SgmRNA levels detected in nasal swabs, throat swabs or BAL fluid in time (days) post Delta virus challenge.

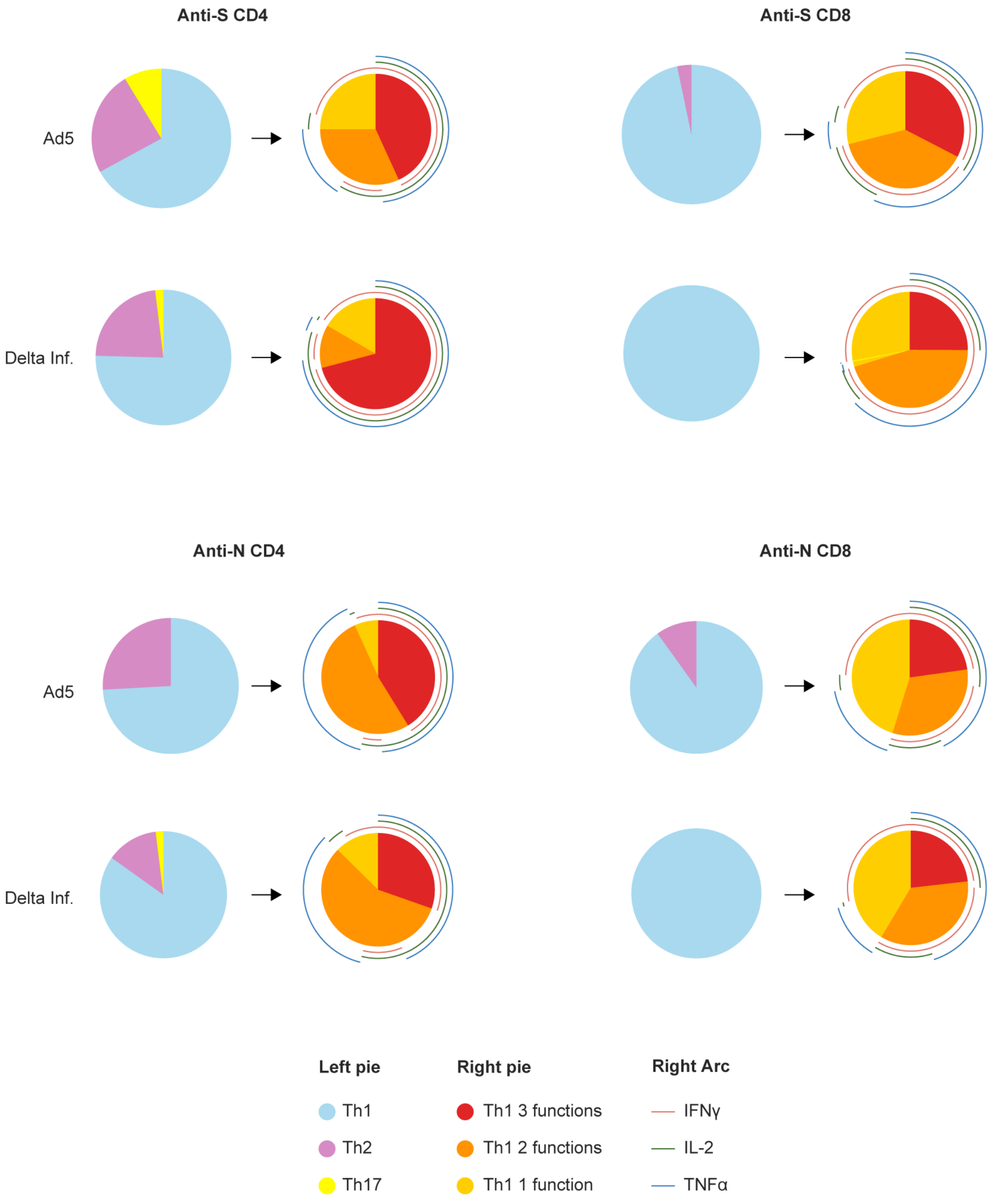

Fig. S5 Polyfunctionality of S- and N-specific CD4 and CD8 T cell responses, measured at week 18. Shown is the relative type 1 (total IFN-γ, IL-2, TNFα), type 2 (IL-4 plus IL-13) and type 17 (IL-17A) cytokine production in the Ad5 boosted and Delta virus infected animals (left pies). Type 1 cytokines are further divided into triple, dual and single IFN-γ, IL-2, TNFα producing cells (right pies), with expression of each of these cytokines shown as an arc around the pie.

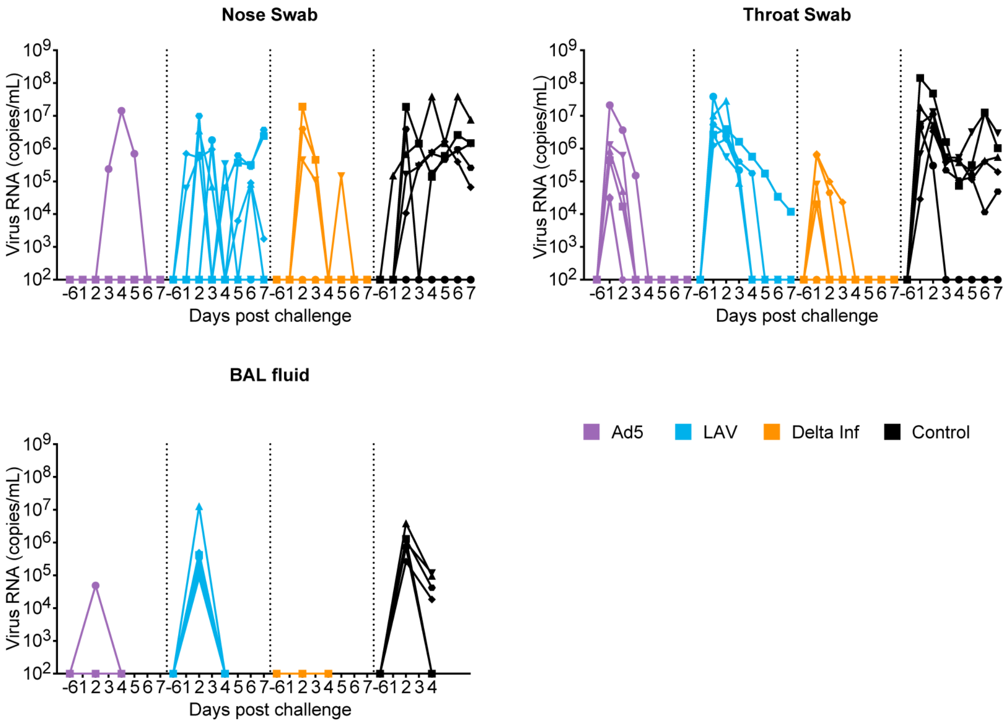

Fig. S6 Genomic RNA in nose, throat and BAL after SARS-CoV-2 Omicron EG.5.1.1 challenge. Shown are the genomic RNA levels (copies/mL) of each individual animal measured over time (days) in nose swabs (upper left graph), throat swabs (upper right graph) and BAL fluid (lower left graph) in the Ad5 boosted (purple), LAV boosted (blue), Delta virus infected (orange) or unvaccinated control animals (black). Challenge was at day 0.

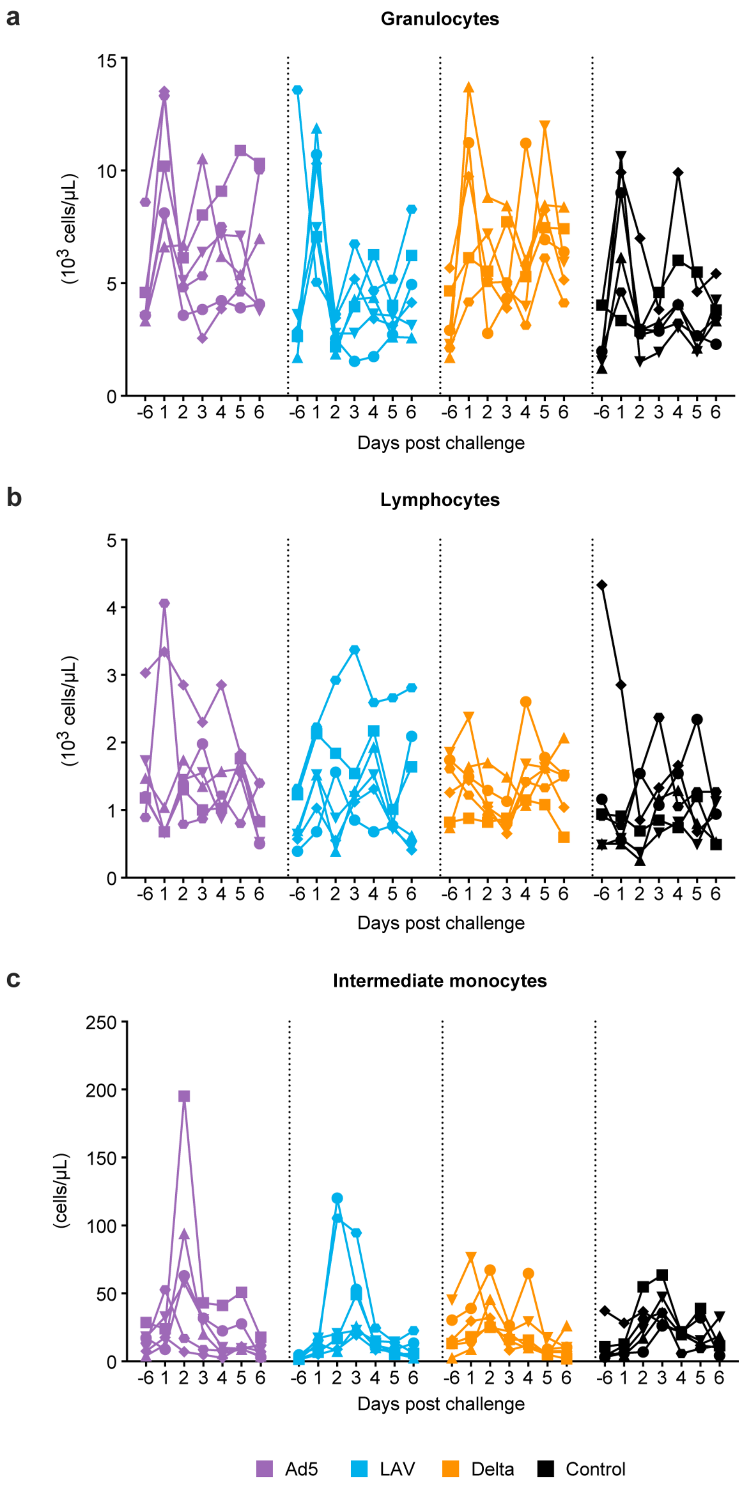

Fig. S7 Peripheral blood leukocyte subsets in time after SARS-CoV-2 Omicron EG.5.1.1 challenge. a neutrophilic granulocyte count (in 10^3 cells/µL), **b** lymphocyte count (in 10^3 cells/µL) and **c** absolute number of intermediate CD16+CD14+ monocytes (cells/µL), for each individual animal in time. Shown are Ad5 boosted (purple), LAV boosted (blue), Delta virus infected (orange) and unvaccinated control animals (black). Challenge was at day 0.

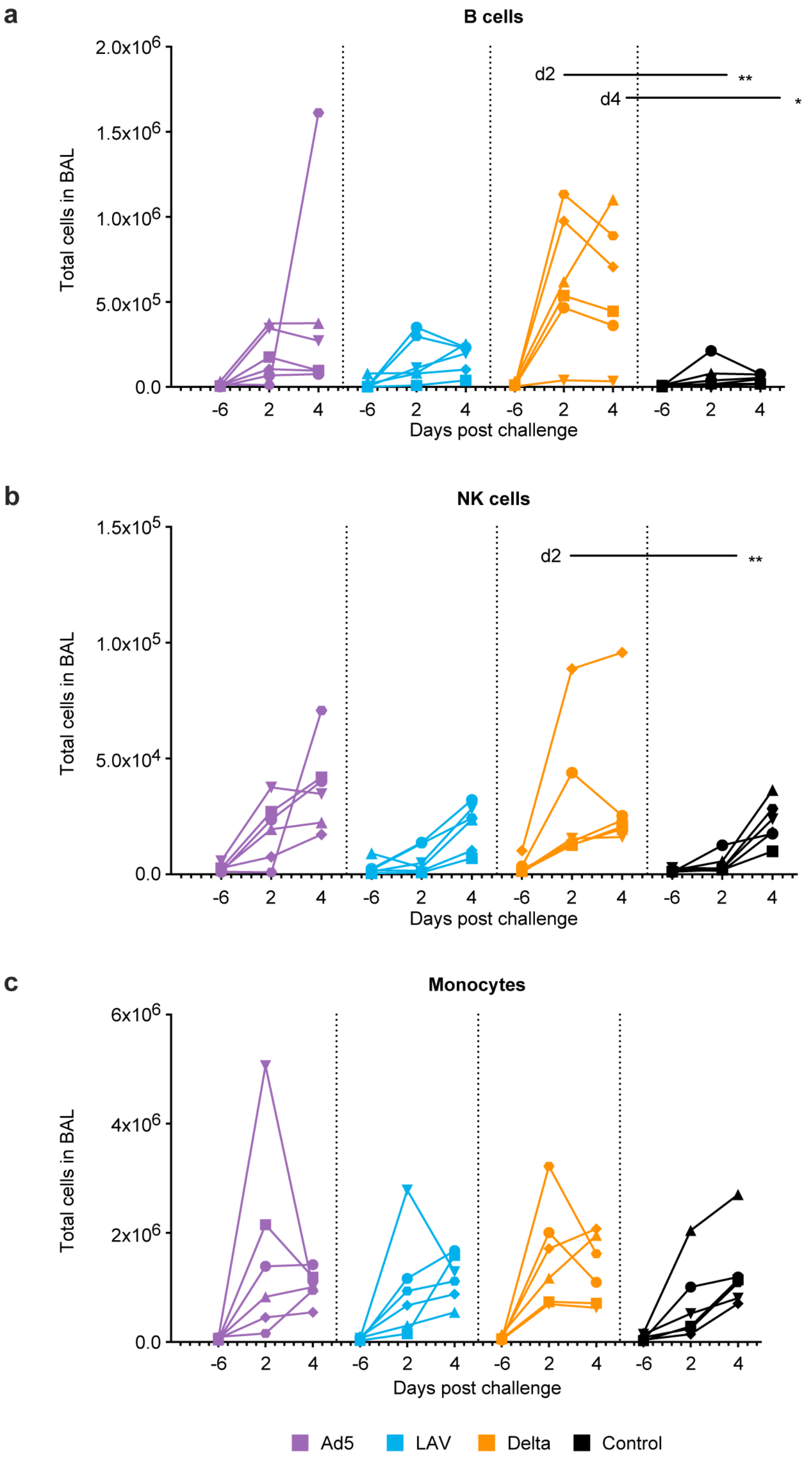

Fig. S8 Changes in B cell, NK cell and monocyte number in BAL fluid after SARS-CoV-2 Omicron EG.5.1.1 challenge. **a** total number of B cells, **b** NK cells, **c** monocytes present in the collected BAL fluid, for each individual animal in time. Shown are Ad5 boosted (purple), LAV boosted (blue), Delta virus infected (orange) and unvaccinated control animals (black). Challenge was at day 0. Statistical differences between the groups were calculated at day 2 (d2) or day 4 (d4) post challenge by Mann-Whitney test; *p=<0.05, **p<0.01.
